# Unveiling novel therapeutic mechanisms of Xinfeng capsule: modulating the ALKBH5–m6A–LINC00968 axis to alleviate oxidative stress-driven NETosis in rheumatoid arthritis

**DOI:** 10.3389/fimmu.2025.1707663

**Published:** 2025-12-17

**Authors:** Yue Sun, Yang Li, Jian Liu

**Affiliations:** 1 Department of Rheumatology, The First Affiliated Hospital of Anhui University of Chinese Medicine, Hefei, Anhui, China; 2 Anhui Provincial Key Laboratory of Applied Basic and Clinical Translational Research in Traditional Chinese Medicine Rheumatology, Hefei, Anhui, China; 3 Institute of Rheumatology, Anhui Academy of Chinese Medicine, Hefei, Anhui, China

**Keywords:** rheumatoid arthritis, Xinfeng capsule, M6A, NETosis, neutrophil, inflammation

## Abstract

**Background:**

Rheumatoid arthritis (RA) is an autoimmune disease characterized by neutrophil infiltration and synovial hyperplasia. Neutrophil extracellular trap (NET) formation and subsequent oxidative stress-inflammation responses play a critical role in RA pathogenesis. Although m6A RNA methylation and the long non-coding RNA LINC00968 are known regulators in RA, their functions in neutrophils and the mechanisms underlying the therapeutic effect of the traditional Chinese medicine formula Xinfeng Capsule (XFC) remain unclear.

**Methods:**

A retrospective clinical study was first conducted involving 2,367 RA patients. Following propensity score matching, the effects of XFC on immune inflammatory markers and liver/kidney safety were evaluated. Subsequently, *in vitro* mechanistic investigations were performed using RT-qPCR, Western blot, MeRIP-qPCR, CCK-8, flow cytometry, immunofluorescence, and ELISA to assess m6A levels, gene expression, cell proliferation, cell cycle, NET formation, and oxidative stress/inflammatory factors. Molecular docking was used to predict the binding affinity between active components of XFC and ALKBH5.

**Results:**

Clinical results demonstrated that XFC significantly improved multiple immunoinflammatory markers in RA patients, including ESR, Hs-CRP, RF, CCP, NLR, and SII, without hepatorenal toxicity. Mechanistically, ALKBH5 was upregulated in the RA microenvironment, leading to reduced m6A methylation and enhanced expression of LINC00968. This axis promoted neutrophil hyperactivation, G1-phase arrest, and NETosis, accompanied by aggravated oxidative stress via the NADP+/NADPH pathway and elevated pro-inflammatory cytokine release (TNF-α, IL-6, and IL-17A). XFC treatment inhibited ALKBH5 activity, increased m6A methylation of LINC00968, and subsequently suppressed neutrophil dysregulation, NET formation, and oxidative stress-inflammation responses. These effects were consistently validated in rescue experiments involving ALKBH5 knockdown and LINC00968 overexpression.

**Conclusion:**

This study unveils a novel ALKBH5–m6A–LINC00968 signaling axis that critically regulates neutrophil hyperactivation and NETosis in RA. XFC attenuates the neutrophil–NET–synovial cell inflammatory cascade by targeting ALKBH5 and promoting m6A methylation of LINC00968, providing an epigenetically regulated therapeutic strategy for RA treatment within a traditional medicine framework.

## 1 Introduction

Rheumatoid arthritis (RA) is a common and refractory autoimmune disease characterized by synovial inflammation and immune-mediated damage to target organs. Its pathogenesis involves dysregulated cell cycle progression, aberrant signaling pathways, oxidative stress, and inflammatory cascades, all of which contribute to disease progression (1). Persistent immune–oxidative stress–inflammatory responses mediated by peripheral blood neutrophils form a vicious cycle that underlies the chronicity of RA (2). Current clinical management emphasizes early and sustained intervention to achieve “treat-to-target” goals (3). Although non-steroidal anti-inflammatory drugs, glucocorticoids, conventional disease-modifying antirheumatic drugs, and newer biological and targeted synthetic agents have shown efficacy, their effectiveness varies significantly among patients due to heterogeneity in treatment response (4). Long-term use also increases the risk of adverse events such as infections, highlighting the need for more effective and safer therapeutic strategies targeting oxidative stress and immune inflammation in RA (5).

Neutrophils, as key effector cells of the innate immune system, play a dual role in RA; they act not only as short-lived first-line defenders against pathogens but also as drivers of autoimmunity in the inflammatory microenvironment. Recent studies highlight the central role of neutrophil extracellular traps (NETs) and NETosis in RA pathogenesis (6). NETs exacerbate autoimmune responses by providing autoantigens (e.g., citrullinated proteins), activating inflammatory signaling, and promoting tissue damage (7). NETosis is a unique form of cell death dependent on reactive oxygen species (ROS). Key initiating events include activation of NADPH oxidase (NOX), leading to superoxide production, followed by peptidylarginine deiminase 4 (PAD4) activation, chromatin decondensation, and nuclear envelope disruption mediated by myeloperoxidase (MPO) and neutrophil elastase (NE) (8). Subsequent release of chromatin decorated with cytoplasmic and granular proteins results in membrane rupture and NET formation (9). NETosis further amplifies inflammation by stimulating the release of cytokines such as tumor necrosis factor-alpha (TNF-α), interleukin-6 (IL-6), interleukin-17A (IL-17A), and interferon-gamma (IFN-γ), contributing to a “cytokine storm” (10). Elevated NETosis has been detected in the synovial fluid and rheumatoid nodules of RA patients (11). Anti-citrullinated H2A/H2B antibodies produced by reactive B cells can specifically recognize NETs and enhance pro-inflammatory gene expression in synovial fibroblasts, underscoring the critical role of NETosis in RA immune inflammation (12).

Epitranscriptomic modifications, especially N6-methyladenosine (m6A) methylation, represent a crucial layer of posttranscriptional regulation mediated dynamically by “writers,” “erasers” (e.g., ALKBH5), and “readers” (13). m6A modification is increasingly implicated in immune cell differentiation, activation, and inflammatory diseases. For instance, m6A methylation in RA synovial tissue promotes TGM2 expression, correlating with activated immune phenotypes, NF-κB activation, antiapoptotic effects, and treatment response (14). Some researchers also had found that the expression of ALKBH5 was increased in RA synovial tissues, collagen-induced arthritis model rats, and RA fibroblast-like synoviocytes (FLSs), and a hypoxic environment increased the expression of ALKBH5 in FLSs. Increased expression of ALKBH5 promoted the proliferation and migration of RA-FLSs and inflammation, whereas these changes showed some correlation with the disease activity (15). Our preliminary work identified long non-coding RNA LINC00968 as a potential player in RA pathology (16). Therefore, we boldly hypothesize that ALKBH5-mediated m6A demethylation of LINC00968 enhances NADP+/NADPH pathway activation, promoting oxidative stress, NETosis, and inflammatory cytokine release, thereby perpetuating immune-inflammatory damage in RA.

Traditional Chinese medicine (TCM) plays a significant role in RA management. Based on the disease mechanism of “spleen deficiency with dampness accumulation and phlegm-stasis obstruction,” our group has developed Xinfeng Capsule (XFC), a Chinese medicinal compound for strengthening the spleen and unblocking collaterals. XFC has been used clinically for years with proven efficacy and safety, and it modulates abnormally expressed lncRNAs (17). Clinical trials demonstrated that XFC significantly improved ACR20, ACR50, and ACR70 responses, with efficacy comparable with leflunomide at 4, 8, and 12 weeks (18). Meta-analyses confirmed that XFC alleviates joint pain, swelling, morning stiffness, and reduces ESR, CRP, and anti-CCP antibody levels (19, 20). Mass spectrometry has identified multiple bioactive components in XFC (21–23). Despite its clinical benefits, the molecular mechanisms underlying XFC’s effects, particularly concerning epitranscriptomic regulation and neutrophil function, remain unclear.

To elucidate how XFC alleviates RA, we propose that XFC modulates immune inflammation by inhibiting oxidative stress and NETosis through targeting ALKBH5-mediated LINC00968 m6A methylation. This study integrates multitiered research strategies encompassing retrospective data mining, bioinformatics prediction, cellular experiments, and molecular docking. While further confirming the efficacy and safety of XFC in clinical analysis, we explored the expression and function of the ALKBH5/LINC00968/m6A axis in neutrophils from RA patients, thereby elucidating the mechanism by which this axis regulates oxidative stress and neutrophil function. *In vitro* experiments validated whether XFC exerts therapeutic effects through the ALKBH5-LINC00968-m6A pathway.

## 2 Materials and methods

### 2.1 Clinical data collection

Clinical data were retrospectively collected from 2,367 RA patients admitted to the Department of Rheumatology at the First Affiliated Hospital of Anhui University of Chinese Medicine. All enrolled patients met the diagnostic criteria established by the American College of Rheumatology (ACR) and the European Alliance of Associations for Rheumatology (EULAR) (24). This study was conducted in accordance with the Declaration of Helsinki and approved by the Ethics Committee of the First Affiliated Hospital of Anhui University of Chinese Medicine (Approval No. 2023AH-52). Due to the retrospective nature of data mining methods, we utilized fully deidentified information that did not impact patient care. All patients provided their written informed consent to participate in this study. Demographic characteristics including age and gender were recorded. Laboratory parameters consisted of neutrophil count, lymphocyte count, monocyte count, platelet count, erythrocyte sedimentation rate (ESR), high-sensitivity C-reactive protein (Hs-CRP), rheumatoid factor (RF), anti-cyclic citrullinated peptide antibody (CCP), immunoglobulins (IgA, IgG, and IgM), complement components (C3 and C4), alanine aminotransferase (ALT), aspartate aminotransferase (AST), creatinine (CREA), and blood urea nitrogen (BUN). The neutrophil-to-lymphocyte ratio (NLR), monocyte-to-lymphocyte ratio (MLR), and systemic immune-inflammation index (SII) were calculated as follows:

1. NLR = neutrophil count (10^9^/L)/lymphocyte count (10^9^/L);
2. MLR = monocyte count (10^9^/L)/lymphocyte count (10^9^/L);
3. SII = [neutrophil count (10^9^/L) × platelet count (10^9^/L)]/lymphocyte count (10^9^/L).

### 2.2 Propensity score matching

To minimize baseline differences between groups, propensity score matching (PSM) was performed. PSM matches each individual with one or more controls sharing similar background characteristics, thereby approximating a randomized study design (25). A 1:1 nearest-neighbor matching algorithm was applied with a caliper width set to 0.20 times the standard deviation of the propensity score.

### 2.3 Association rule analysis

Association rule analysis was conducted using the Apriori algorithm in IBM SPSS Modeler 18.0 to identify relationships between XFC treatment and improvements in clinical laboratory parameters. XFC treatment was labeled as “T” and non-XFC treatment as “F”. A decrease in laboratory values after treatment was defined as “T”, whereas stable or increased values were defined as “F”. Support, confidence, and lift values were calculated as described in previous studies (26).

### 2.4 Random walk model

A random walk model was developed using ORACLE 10g to track longitudinal changes in laboratory indicators among RA patients, enabling dynamic monitoring of long-term treatment effects of XFC. The computational procedure followed established methodologies (26).

### 2.5 Cell isolation and culture

Peripheral blood polymorphonuclear leukocytes (PMNs) were collected and isolated from hospitalized RA patients. This study likewise obtained informed consent from both the hospital ethics committee and the patients (Approval No. 2023AH-31). Specifically, peripheral venous blood (5 mL) was first collected using EDTA anticoagulant tubes and mixed 1:1 with PBS. Subsequently, PMNs were isolated via Percoll continuous density gradient centrifugation. Cell purity (≥95%) was confirmed by Wright–Giemsa and trypan blue staining. Primary human fibroblast-like synoviocytes (FLSs) were purchased from iCell Bioscience (Cat#: HUM-iCell-008a) and isolated, identified, and cultured according to Sun et al. (21). For coculture experiments, FLSs and PMNs were seeded in the lower and upper chambers of Transwell plates, respectively. Cells from the lower chamber were collected for subsequent analysis.

### 2.6 Cell transfection

Overexpression plasmids (pcDNA3.1) for ALKBH5 and LINC00968, along with ALKBH5-targeting siRNA, were constructed using Lipofectamine 2000 (Invitrogen, Carlsbad, CA, USA) and transfected into RA-PMNs. siRNA sequences are listed in **Table 1**.

**Table 1:** Primer sequences of RT-qPCR for genes.

Gene	Amplicon size (bp)	Forward primer (5'→3')	Reverse primer (5'→3')
si-ALKBH5-1	/	GCUGCAAGUUCCAGUUCAAGC	UUGAACUGGAACUUGCAGCCG
si-ALKBH5-2	/	GCUUCAGCUCUGAGAACUACU	UAGUUCUCAGAGCUGAAGCUA
si-ALKBH5-3	/	GGACCUAGGUUCUCAUAUUCU	AAUAUGAGAACCUAGGUCCUG
ALKBH5	81	GGAATGTCTTCTTGTCAGCC	AAGTGGTGGTATCCTGGTTG
LINC00968	75	TGGTCCATTTGGATGGGAAA	TGATACCGGTGACCACATTT
DNase I	190	TTTTACAGCAGCAAGAAACC	CTCATCCTGAGATGATGGTG
NOX	192	CTCGGAGGTCTGAAAAATGA	ATGAACAGTTCTCCTTGGTC
HO-1	175	AAAAAGATTGCCCAGAAAGC	CAGTCTTGGCCTCTTCTATC
β-actin	96	CCCTGGAGAAGAGCTACGAG	GGAAGGAAGGCTGGAAGAGT

### 2.7 Preparation of XFC-containing serum

The XFC used in this study (medical approval number: Z20050062, batch number: 20221017) was purchased from the Pharmacy Department of The First Affiliated Hospital of Anhui University of Chinese Medicine. The capsules were stored in their original packaging at room temperature in a dry and dark place, in accordance with the manufacturer’s recommendations, until use. The quality and composition of XFC, including the identity of active components such as calycosin, calycosin-7-glucoside, and formononetin, were rigorously controlled and consistent with previously established chromatographic fingerprints (23). A total of 20 male Sprague-Dawley rats were randomly divided into normal serum and drug-containing serum groups. The drug-containing group received XFC suspension (0.648 g/100 g/day) via gavage, whereas the control group received an equal volume of 0.9% saline. After 1 week of administration, blood was collected from the abdominal aorta under anesthesia induced by sodium pentobarbital (50 mg/kg). Serum was separated by centrifugation (3,000 rpm, 15 min), inactivated at 56 °C for 30 min, filter-sterilized, and stored at −80 °C. All animal procedures were approved by the Animal Ethics Committee of Anhui University of Chinese Medicine (AHUCM-rats-2023020).

### 2.8 Colorimetric detection of total m6A levels

Total m6A levels in extracted RNA were measured using an m6A RNA Methylation Quantification Kit according to the manufacturer’s instructions. Total RNA was bound to wells using a high-affinity RNA binding solution. m6A was detected using capture and detection antibodies, and quantification was performed by measuring optical density (OD) at 450 nm.

### 2.9 Real-time quantitative polymerase chain reaction

Total RNA was extracted using TRIzol reagent and reverse-transcribed into cDNA. qPCR was performed using Taq SYBR Green qPCR Premix (Universal). β-Actin served as the internal control, and the 2^−ΔΔCt method was used to calculate relative mRNA expression. All primer sequences are listed in **Table 1**.

### 2.10 M6A methylation immunoprecipitation-qPCR

Total RNA was extracted and fragmented from RA-PMNs. Precoupled magnetic beads with m6A antibody were used to immunoprecipitate m6A-modified RNA fragments. After elution, RNA was reverse-transcribed and amplified using primers specific to m6A modification sites.

### 2.11 Western blot

Cells were lysed in RIPA buffer, and total protein was extracted. Proteins were denatured, separated by SDS-PAGE, transferred to membranes, and blocked. Membranes were incubated with primary antibodies against ALKBH5 (1:1,000, ab195377, Abcam, UK), NOX (1:2,000, bsm-52390R, Bioss, China), and HO-1 (1:500, SC-136960, Santa Cruz, US) overnight at 4°C, followed by the addition of HRP-labeled secondary antibody diluted 1:20,000. Bands were visualized using chemiluminescence and analyzed with ImageJ software. GAPDH (1:2000, TA-08, ZSBiO, China) served as the loading control.

### 2.12 Actinomycin D assay

Cells were treated with 5 μg/mL actinomycin D and harvested at 0, 2, 4, 6, 8, and 10 h. RNA was extracted, and LINC00968 expression was assessed via real-time quantitative polymerase chain reaction (RT-qPCR). Experimental groups included NC-PMN, RA-PMN, RA-PMN+OE-NC, RA-PMN+OE-ALKBH5, RA-PMN+si-NC, and RA-PMN+si-ALKBH5.

### 2.13 CCK-8 assay

Cell suspensions were prepared and incubated overnight. CCK-8 reagent (10 μL) was added, and absorbance was measured at 450 nm.

### 2.14 Flow cytometry

Cells were washed with cold PBS, digested with trypsin, and centrifuged at 2,000 rpm for 5 min. Pellets were resuspended and fixed in ethanol at 4°C overnight. After washing, cells were stained with RI/RNase Staining Buffer and analyzed by flow cytometry.

### 2.15 Immunofluorescence staining

Neutrophils were fixed, permeabilized, and blocked. Cells were incubated with anti-NE antibody (1:100), followed by fluorescent secondary antibody and tyramide signal amplification (TSA). After antibody elution, the same procedure was repeated with anti-MPO antibody (1:300). Nuclei were counterstained with DAPI. Images were acquired using a confocal microscope and analyzed with ImageJ.

### 2.16 Enzyme-linked immunosorbent assay

Levels of NOX, HIF-1α, HO-1, TNF-α, IL-6, and IL-17A in cell supernatants were measured using commercial ELISA kits (JYM0646Ra, JYM0654Ra, and JYM0647Ra; Wuhan Genmei Technology Co., Ltd., China) according to the manufacturer’s instructions.

### 2.17 Molecular docking

Three active components of XFC (calycosin, calycosin-7-glucoside, and formononetin) identified via mass spectrometry were retrieved from the TCSMP database (23). The ALKBH5 structure was obtained from the PDB database (https://www.rcsb.org). Docking simulations were performed using AutoDock Vina and PyMOL.

### 2.18 Statistical analysis

Data were analyzed using SPSS 26.0 and GraphPad Prism 9.6. Normally distributed continuous variables were compared using t-tests (two groups) or one-way ANOVA with Sidak’s multiple comparisons test (multiple groups). Non-normally distributed data were analyzed using non-parametric tests. Statistical significance was set at P < 0.05.

## 3 Results

### 3.1 XFC effectively improves immunoinflammatory indicators in RA patients without hepatorenal toxicity risk

**Figure 1:**
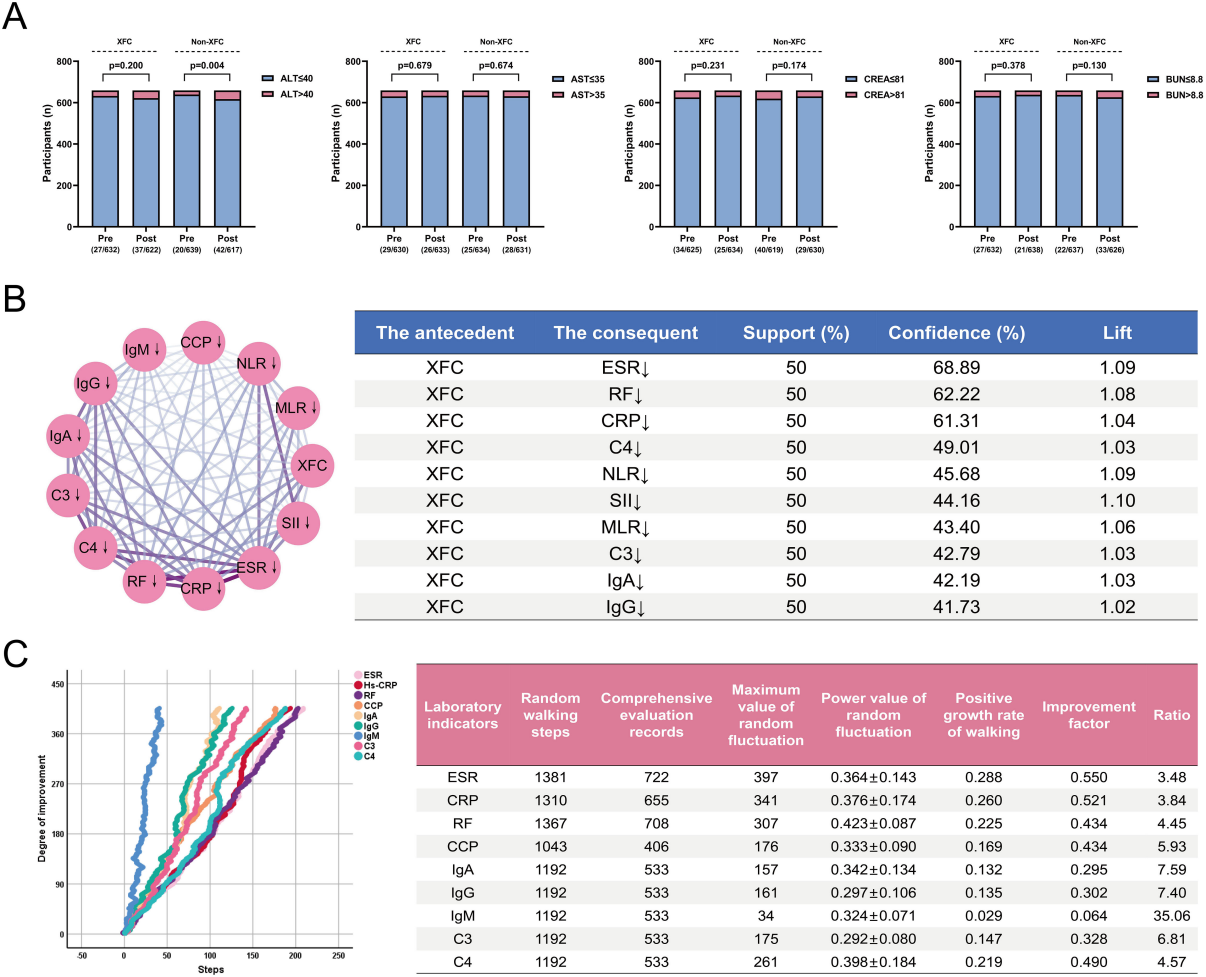
XFC effectively improves the immune inflammatory indicators of RA patients without liver or kidney risks. **(A)** Distribution ratio of liver and kidney function indicators before and after treatment in the XFC group and the non-XFC group. **(B)** Based on the Apriori algorithm, the association rule analysis network graph was conducted, and the association pattern between XFC processing and the improvement of immune inflammatory indicators was obtained. **(C)** A random walk model regarding the relationship between XFC treatment and the improvement of immune inflammatory indicators.

To evaluate the clinical efficacy of XFC on immunoinflammatory parameters in RA patients, we retrospectively enrolled 2,367 RA patients, including 1,210 who received XFC treatment. Following a 1:1 propensity score matching (PSM) protocol, a final cohort of 1,318 patients was generated, with no significant differences in most baseline variables between the two groups (all P > 0.05; **Table 2**). As shown in **Table 3**, both groups exhibited significant reductions in ESR, Hs-CRP, RF, CCP, IgA, IgG, C3, and C4 levels after treatment (all P < 0.001). Notably, the XFC treatment group showed more pronounced improvements in NLR, SII, ESR, RF, and CCP levels compared with controls (all P < 0.05). Furthermore, XFC significantly reduced NLR, MLR, SII, and IgM values (all P < 0.05). Importantly, no statistically significant changes were observed in liver and kidney function markers—ALT, AST, CREA, and BUN—before and after XFC treatment (all P > 0.05; **Figure 1A**). Association rule analysis based on the Apriori algorithm revealed strong correlations between XFC treatment and improvements in ESR, RF, Hs-CRP, C4, NLR, SII, MLR, C3, IgA, and IgG (**Figure 1B**). To further assess the long-term efficacy of XFC, a random walk model was applied. The results demonstrated that prolonged XFC treatment was associated with progressively greater improvements in ESR, Hs-CRP, RF, CCP, IgA, IgG, IgM, C3, and C4 levels (**Figure 1C**), indicating a sustained therapeutic effect on immunoinflammatory responses in RA patients. Collectively, these observational findings demonstrate that XFC effectively ameliorates immunoinflammatory activity in RA patients without conferring hepatorenal toxicity.

**Table 2:** Baseline characteristics of patients who used and did not use XFC before and after PSM were analyzed.

Variables	Before PSM				After PSM			
	Overall	Non-XFC	XFC	P value	Overall	Non-XFC	XFC	P value
Participants	2,367	1,157	1,210		1,318	659	659	
Gender, n(%)				<0.001				0.170
Male	445(18.80)	183(15.82)	262(21.65)		233(17.68)	107(16.24)	126(19.12)	
Female	1,922(81.20)	974(84.18)	948(78.35)		1,085(82.32)	522(79.21)	533(80.88)	
Age, year	58(51, 68)	55(48, 66)	62(53, 70)	<0.001	63(53, 69)	63(53, 70)	62(53, 69)	0.176
NLR	2.83(2.02, 4.07)	2.84(1.98, 4.18)	2.83(2.03, 3.99)	0.825	2.82(1.99, 4.05)	2.84(2.01, 4.11)	2.80(1.97,3.99)	0.617
MLR	0.29(0.22, 0.39)	0.29(0.22, 0.39)	0.29(0.22, 0.39)	0.611	0.30(0.22, 0.39)	0.30(0.22, 0.39)	0.29(0.22, 0.38)	0.262
SII	696.16(423.97, 1,113.89)	679.22(422.39, 1,106.65)	714.07(428.13, 1,118.61)	0.418	677.32(419.37, 1,079.64)	667.74(413.09, 1,061.51)	686.16(420.27, 1,093.31)	0.598
ESR, mm/h	41.00(23.00, 66.00)	36.00(21.00, 59.00)	46.00(26.00, 69.00)	<0.001	40.00(23.00, 65.00)	37.00(23.00, 64.00)	44.00(24.00, 66.00)	0.090
Hs-CRP, mg/L	13.60(3.56, 38.64)	10.48(2.94, 34.25)	17.56(4.56, 43.29)	<0.001	12.53(3.08, 36.57)	11.24(2.95, 36.02)	13.36(3.19, 36.66)	0.169
RF, KIU/L	95.70(34.70, 240.90)	92.70(36.35, 229.60)	97.50(33.80, 248.45)	0.931	89.80(33.88, 238.80)	88.10(33.80, 218.10)	94.60(33.90, 258.80)	0.428
CCP, U/ml	106.00(25.00, 314.00)	82.10(17.35, 242.5)	126.40(26.88, 370.90)	<0.001	84.00(16.78, 236.25)	73.50(17.20,235.00)	97.00(15.80, 241.00)	0.348
IgA, g/L	2.65(1.96, 3.53)	2.60(1.91, 3.51)	2.68(2.01, 3.55)	0.057	2.73(2.00, 3.55)	2.71(1.96, 3.51)	2.76(2.06, 3.56)	0.148
IgG, g/L	11.62(9.31, 14.72)	11.04(8.85, 13.79)	12.30(9.76, 15.55)	<0.001	11.27(9.02, 14.29)	11.04(8.70, 14.16)	11.51(9.20, 14.49)	0.035
IgM, g/L	1.26(0.91, 1.70)	1.31(0.94, 1.79)	1.21(0.89, 1.63)	0.001	1.25(0.89, 1.68)	1.25(0.87, 1.66)	1.25(0.93, 1.69)	0.432
C3, g/L	1.25(1.07, 1.71)	1.18(1.03, 1.33)	1.45(1.15, 110.00)	<0.001	1.17(1.03, 1.33)	1.16(1.01, 1.32)	1.18(1.04, 1.34)	0.150
C4, g/L	0.32(0.24, 0.57)	0.28(0.22, 0.35)	0.45(0.27, 23.80)	<0.001	0.29(0.23, 0.36)	0.29(0.23, 0.36)	0.29(0.23, 0.37)	0.834
ALT, U/L	13(9, 19)	13(10, 20)	13(9, 18)	0.005	12(9, 18)	12(9, 18)	13(9, 18)	0.685
AST, U/L	17.00(14.00, 21.00)	17.00(14.00, 21.50)	17(13.00, 21.00)	0.002	17.00(14.00, 21.00)	17.00(14.00, 21.00)	17.00(14.00, 21.00)	0.911
CREA, µmol/L	50.40(43.20, 59.80)	50.40(43.25, 59.15)	50.50(43.20, 60.40)	0.537	50.45(43.20, 60.03)	49.70(43.00, 59.40)	51.00(43.50, 61.00)	0.119
BUN, mmol/L	4.78(3.87, 5.86)	4.67(3.76, 5.79)	4.90(3.97, 5.90)	0.003	4.80(3.87, 5.86)	4.73(3.83, 5.89)	4.86(3.89, 5.83)	0.473

Categorical variables were shown as n (percent, %). Continuous variables were shown as median (interquartile range, IQR). P values for differences between groups were derived using a Pearson’s chi-squared test or Mann–Whitney U test.

**Table 3:** Comparison of immune inflammatory indicators before and after treatment in patients with and without XFC.

Indicator	Non-XFC			XFC			P value
	Pretreatment	Posttreatment	P value	Pretreatment	Posttreatment	P value	
NLR	2.84(2.01, 4.11)	2.86(1.95, 4.01)	0.701	2.80(1.97, 3.99)	2.62(1.92, 3.77)	<0.001	0.019
MLR	0.30(0.22, 0.39)	0.30(0.23, 0.39)	0.778	0.29(0.22, 0.38)	0.28(0.21, 0.37)	0.013	0.077
SII	667.74(413.09, 1061.51)	667.51(441.35, 1043.64)	0.442	686.16(420.27, 1093.31)	652.87(417.60, 1041.96)	0.003	0.009
ESR, mm/h	37.00(23.00, 64.00)	29(16.00, 43.00)	<0.001	44.00(24.00, 66.00)	27.00(16.00, 44.00)	<0.001	0.002
Hs-CRP, mg/L	11.24(2.95, 36.02)	2.62(0.74, 9.32)	<0.001	13.36(3.19, 36.66)	2.70(0.86, 11.82)	<0.001	0.220
RF, KIU/L	88.10(33.80, 218.10)	78.6(30.60, 199.30)	<0.001	94.60(33.90, 258.80)	83.2(30.10, 220.50)	<0.001	0.002
CCP, U/ml	73.50(17.20, 235.00)	68.20(15.60, 215.00)	<0.001	97.00(15.80, 241.00)	80.70(13.60, 204.00)	<0.001	0.010
IgA, g/L	2.71(1.96, 3.51)	2.58(1.88, 3.33)	<0.001	2.76(2.06, 3.56)	2.64(1.98, 3.44)	<0.001	0.372
IgG, g/L	11.04(8.70, 14.16)	10.80(8.56, 13.21)	<0.001	11.51(9.20, 14.49)	10.95(8.86, 13.50)	<0.001	0.492
IgM, g/L	1.25(0.87, 1.66)	1.23(0.86, 1.68)	0.450	1.25(0.93, 1.69)	1.23(0.91, 1.67)	0.033	0.285
C3, g/L	1.16(1.01, 1.32)	1.11(0.97, 1.26)	<0.001	1.18(1.04, 1.34)	1.12(0.98, 1.28)	<0.001	0.451
C4, g/L	0.29(0.23, 0.36)	0.26(0.19, 0.32)	<0.001	0.29(0.23, 0.37)	0.26(0.19, 0.33)	<0.001	0.385

Continuous variables were shown as median (Interquartile range, IQR). P values for the within-group differences were performed by Wilcoxon signed rank test. P values for differences between groups were derived using the Mann–Whitney U test.

### 3.2 ALKBH5 is upregulated in RA and mediates m6A modification stability of LINC00968

**Figure 2:**
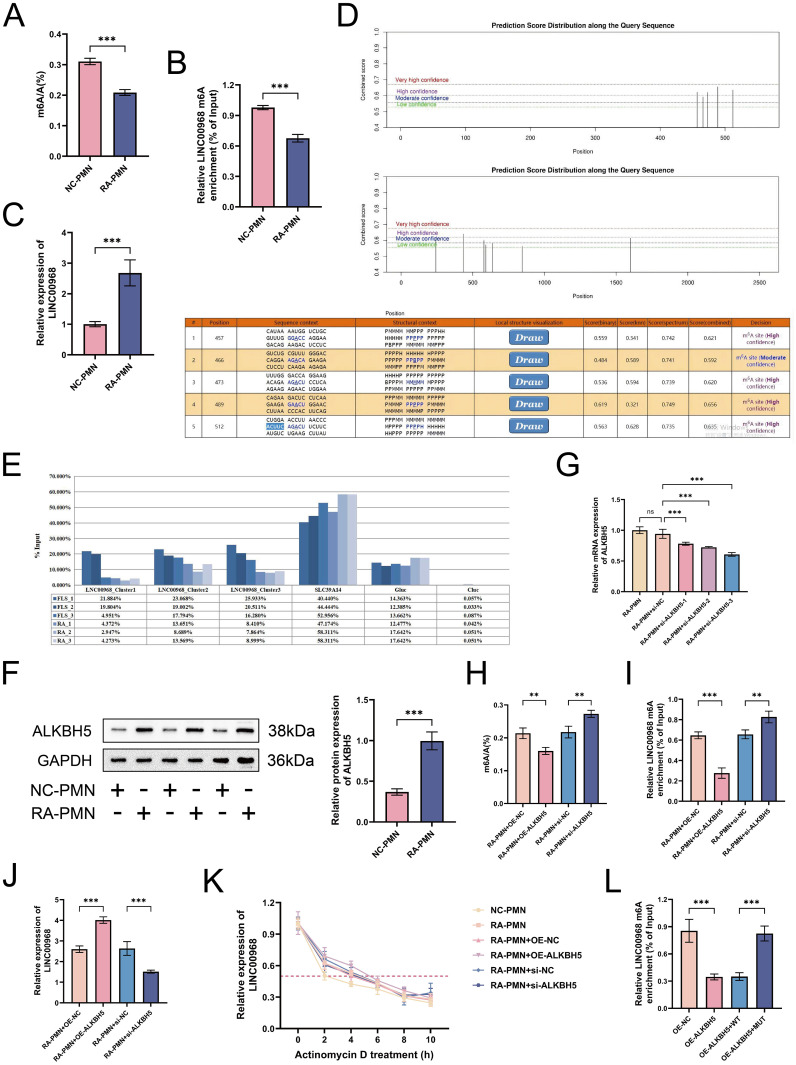
ALKBH5 is upregulated in RA and mediates m6A modification stability of LINC00968. **(A)** Total m6A levels in neutrophils from RA patients (RA-PMNs). **(B)** m6A modification levels of LINC00968 in RA-PMNs measured by MeRIP-qPCR. **(C)** Prediction of potential m6A methylation sites in LINC00968 using the SRAMP online database. **(D)** Relative expression of LINC00968 detected by RT-qPCR. **(E)** MeRIP-qPCR analysis of m6A-enriched regions on LINC00968. **(F)** Protein expression levels of ALKBH5 in RA-PMNs assessed by Western blot. **(G)** Transfection efficiency of three distinct ALKBH5-targeting siRNAs evaluated by RT-qPCR. **(H)** Changes in global m6A levels after ALKBH5 overexpression or knockdown. **(I)** m6A modification levels of LINC00968 following ALKBH5 modulation using MeRIP-qPCR. **(J)** LINC00968 expression after ALKBH5 overexpression or knockdown analyzed via RT-qPCR. **(K)** Effect of ALKBH5 silencing or overexpression on LINC00968 RNA stability using actinomycin D assay. **(L)** Impact of site-directed mutagenesis of m6A motifs on LINC00968 m6A levels upon ALKBH5 overexpression. All data are expressed as mean ± standard deviation. **p < 0.01, ***p < 0.001; ns, not significant.

We next investigated alterations in m6A modification and the underlying regulatory mechanisms in the model group. Experimental results revealed that both the total m6A level and the m6A modification level of LINC00968 were significantly decreased in the model group compared with the control group (**Figures 2A, B**), whereas the expression level of LINC00968 was markedly increased (**Figure 2C**). Using the online database SRAMP (http://www.cuilab.cn/sramp), four high-probability m6A sites were predicted along the full length of LINC00968 (**Figure 2D**). To validate this prediction, we performed M6A methylation immunoprecipitation-qPCR (MeRIP-qPCR) and identified a hotspot region of m6A modification on the LINC00968 transcript (**Figure 2E**). Subsequently, we explored the upstream regulatory mechanisms of methylation. Western blot analysis showed that the protein expression level of the demethylase ALKBH5 was significantly elevated in the model group (**Figure 2F**). To confirm the demethylation-mediated role of ALKBH5 in the RA microenvironment, we performed both overexpression and knockdown experiments in RA-PMN cells. Among three different siRNAs tested, siRNA-3 demonstrated the most efficient knockdown efficacy (**Figure 2G**). As expected, compared with the NC group, overexpression of ALKBH5 significantly suppressed both global m6A levels and LINC00968-specific m6A modification, while upregulating LINC00968 expression. Conversely, knockdown of ALKBH5 produced the opposite effects (all P < 0.01; **Figures 2H–J**). Further data confirmed that ALKBH5 overexpression markedly extended the half-life of LINC00968, whereas ALKBH5 silencing promoted its degradation (**Figure 2K**). Additionally, mutagenesis experiments provided further evidence for the regulatory role of ALKBH5. Results showed that the reduction in LINC00968 m6A levels induced by ALKBH5 overexpression was effectively reversed in the mutant (MUT) group compared with the wild-type (MT) group (**Figure 2L**). Collectively, these findings suggest that ALKBH5 promotes the upregulation of LINC00968 in RA by mediating the stability of its m6A modification.

### 3.3 ALKBH5 promotes abnormal survival and NETosis in RA-PMNs

**Figure 3:**
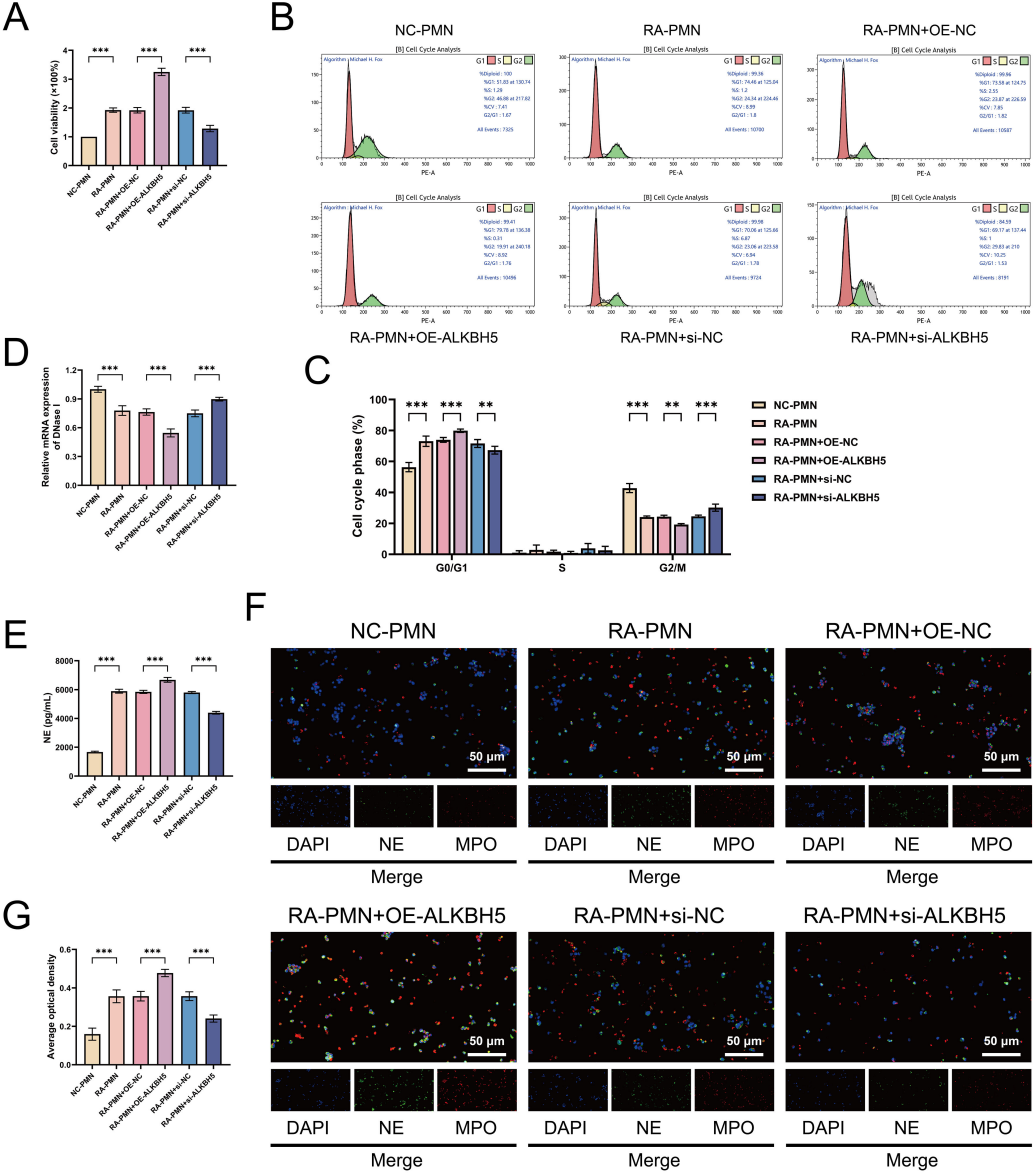
ALKBH5 promotes proliferation, cell cycle delay, and NET formation in RA-PMNs. **(A)** Cell viability of RA-PMNs after ALKBH5 overexpression or knockdown, measured by CCK-8 assay. **(B, C)** Cell cycle distribution of RA-PMNs following ALKBH5 modulation, analyzed by flow cytometry. **(D)** mRNA expression level of DNase I after ALKBH5 overexpression or knockdown, detected via RT-qPCR. **(E)** Expression level of neutrophil elastase (NE) following ALKBH5 modulation, assessed by ELISA. **(F, G)** Subcellular localization and expression of NE (green) and myeloperoxidase (MPO, red) after ALKBH5 overexpression or knockdown, visualized by immunofluorescence staining. All data are presented as mean ± standard deviation. **p < 0.01, ***p < 0.001.

We subsequently investigated the regulatory role of ALKBH5 in RA-PMN viability and cellular processes. CCK-8 assays revealed significantly elevated cell viability in RA-PMNs compared with the NC group. Overexpression of ALKBH5 further promoted cell survival, whereas its knockdown markedly reduced cell viability (**Figure 3A**). The analysis strategy of flow cytometry is presented in **Supplementary Figure 1**. The results showed an increased proportion of cells in the G1 phase and a decreased proportion in the G2 phase in the RA-PMN group relative to the NC group. Notably, ALKBH5 overexpression led to a significant increase in G1-phase cells and a corresponding decrease in G2-phase cells compared with the OE-NC group. In contrast, ALKBH5 knockdown produced the opposite effects (**Figures 3B, C**). These findings indicate that ALKBH5 contributes to abnormal survival of RA-PMNs by both delaying cell progression and enhancing cellular metabolic activity. Furthermore, we examined the release of NETs. Previous studies have reported that DNase I, a specific NET-degrading enzyme, cleaves and fragments DNA, thereby disrupting NET integrity (27). RT-qPCR analysis demonstrated that DNase I expression was significantly downregulated in RA-PMNs compared with the NC group. Overexpression of ALKBH5 further reduced DNase I mRNA levels, whereas ALKBH5 inhibition markedly increased its expression (**Figure 3D**). ELISA results indicated that NE levels were significantly elevated in RA-PMNs relative to the NC group. ALKBH5 overexpression further increased NE expression, whereas ALKBH5 knockdown reversed this trend (**Figure 3E**). Additionally, immunofluorescence staining was performed to detect the expression and co-localization of NE and MPO, markers of NET formation. The results showed a lower rate of NE/MPO co-positive staining in the nuclei of RA-PMNs compared with the NC group. Overexpression of ALKBH5 significantly increased the co-localization of NE and MPO, whereas ALKBH5 silencing reduced it (**Figures 3F, G**), indicating that ALKBH5 expression influences NET formation. Those findings demonstrate that ALKBH5 promotes abnormal neutrophil survival and facilitates NETosis in the context of RA.

### 3.4 ALKBH5 promotes oxidative stress-inflammatory response in RA-PMNs and is highly associated with NET formation

**Figure 4:**
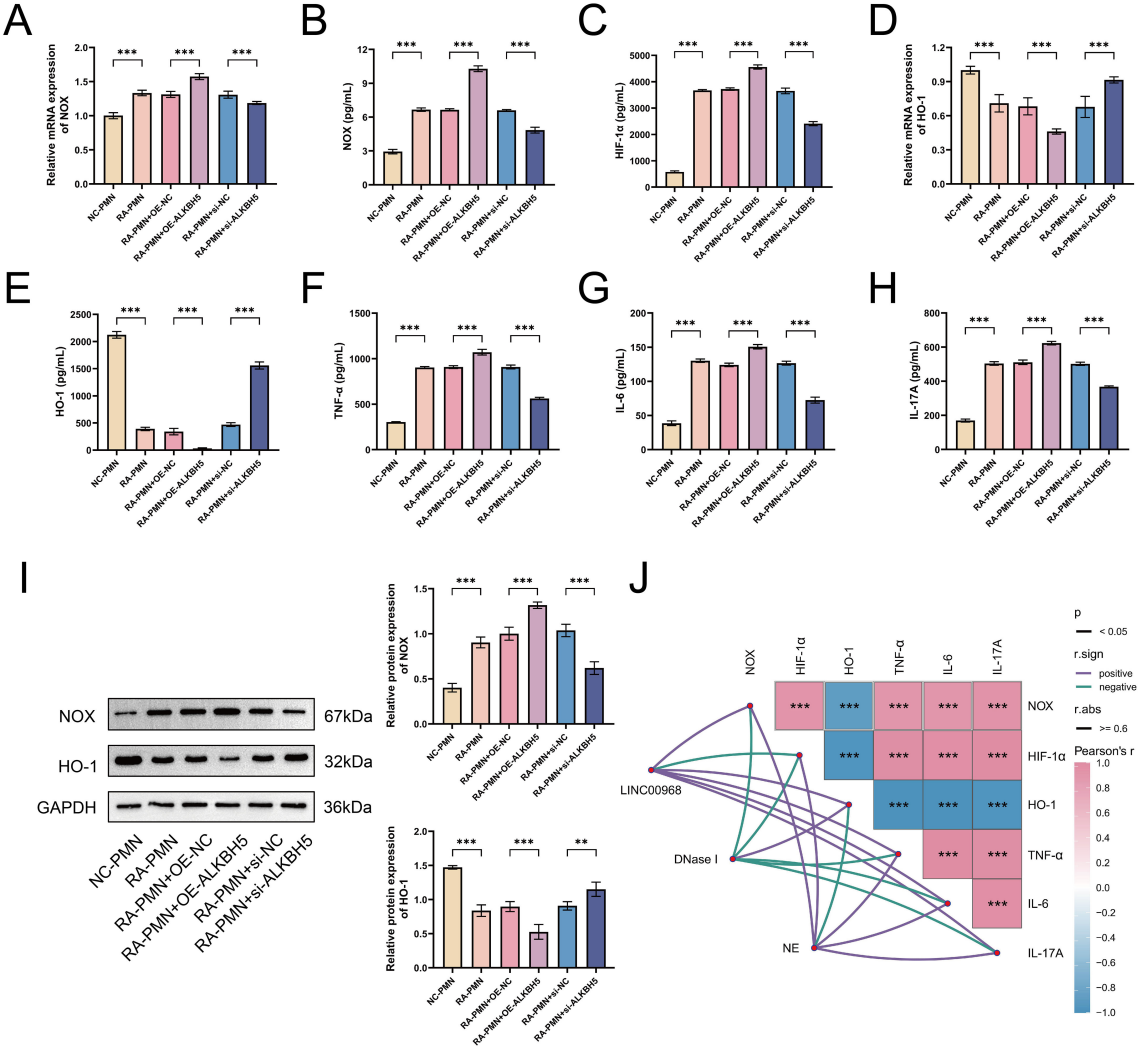
ALKBH5 promotes oxidative stress-inflammatory response in RA-PMNs and is highly associated with NET formation. **(A)** mRNA expression level of NOX after ALKBH5 overexpression or knockdown, detected by RT-qPCR. **(B)** Protein expression level of NOX following ALKBH5 modulation, measured by ELISA. **(C)** mRNA expression level of HIF-1α after ALKBH5 overexpression or knockdown, analyzed via RT-qPCR. **(D)** mRNA expression level of HO-1 after ALKBH5 modulation, detected by RT-qPCR. **(E)** Protein expression level of HO-1 following ALKBH5 overexpression or knockdown, assessed by ELISA. **(F–H)** Secretion levels of TNF-α, IL-6, and IL-17A after ALKBH5 modulation, measured by ELISA. **(I)** Protein expression levels of NOX and HO-1 following ALKBH5 overexpression or knockdown, analyzed by Western blot. **(J)** Correlation analysis among LINC00968, DNase I, NE, and oxidative stress-inflammatory markers (NOX, HIF-1α, HO-1, TNF-α, IL-6, and IL-17A) using Pearson’s test. All data are presented as mean ± standard deviation. **p < 0.01, ***p < 0.001.

Previous studies have indicated that ROS production, primarily triggered by NOX, serves as an initial event in NET formation within the inflammatory milieu of RA (28). We sought to evaluate whether ALKBH5 influences the oxidative stress-inflammatory response in RA-PMNs. Our data revealed that NOX expression was significantly elevated in RA-PMNs compared with the NC group. Overexpression of ALKBH5 further enhanced NOX expression, whereas its knockdown produced the opposite effect (**Figures 4A, B**). RT-qPCR analysis indicated that HIF-1α mRNA levels were markedly higher in RA-PMNs than in the NC group. ALKBH5 overexpression further increased HIF-1α expression, whereas ALKBH5 inhibition reduced it (**Figure 4C**). Additionally, HO-1 expression was downregulated in RA-PMNs. This decrease was further exacerbated by ALKBH5 overexpression and partially reversed upon ALKBH5 knockdown (**Figures 4D, E**). ELISA results demonstrated that upregulation of ALKBH5 increased the secretion of pro-inflammatory cytokines, including TNF-α, IL-6, and IL-17A. Conversely, inhibition of ALKBH5 led to a reduction in these cytokines (**Figures 4F–H**). Western blot analysis further confirmed that ALKBH5 overexpression promoted NOX protein expression and suppressed HO-1 expression. Silencing ALKBH5 yielded opposite protein expression trends (**Figure 4I**). To explore the statistical relationships among these parameters, Pearson correlation analysis was performed. The results showed that the expression levels of both LINC00968 and NE were strongly positively correlated with NOX, HIF-1α, TNF-α, IL-6, and IL-17A, and negatively correlated with HO-1. In contrast, DNase I expression was positively correlated with HO-1 and negatively correlated with NOX, HIF-1α, TNF-α, IL-6, and IL-17A (**Figure 4J**). Collectively, these findings demonstrate that ALKBH5 exacerbates oxidative stress and inflammatory responses in RA-PMNs and is closely associated with NET formation.

### 3.5 ALKBH5 regulates RA-PMN abnormal survival and NETosis by mediating m6A modification of LINC00968

**Figure 5:**
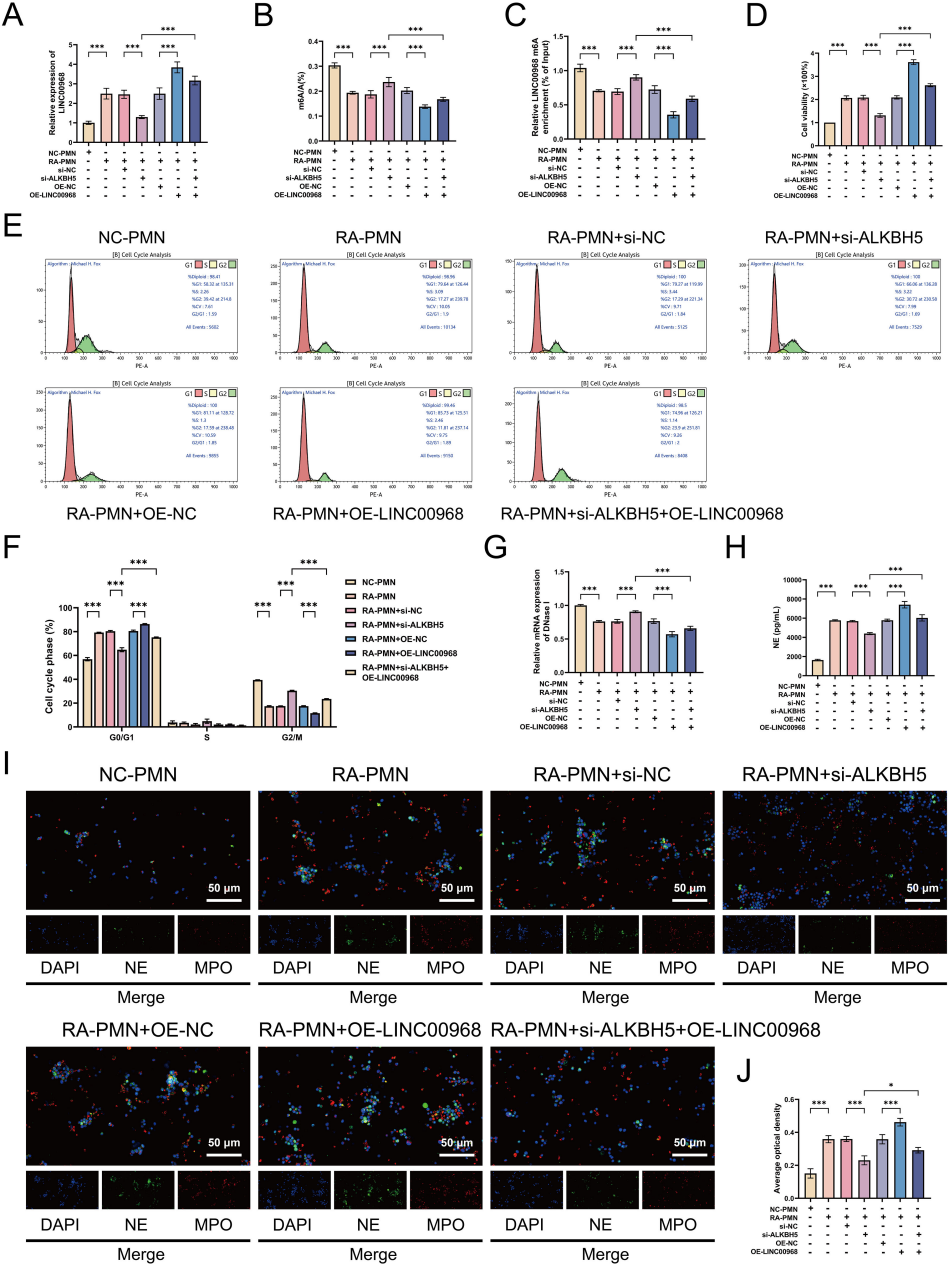
ALKBH5 regulates RA-PMN proliferation, cell cycle arrest, and NET formation via m6A modification of LINC00968. **(A)** LINC00968 expression following ALKBH5 knockdown and LINC00968 overexpression was detected by RT-qPCR. **(B)** m6A levels of LINC00968 were measured using MeRIP-qPCR after ALKBH5 silencing and LINC00968 overexpression. **(C)** Effects of ALKBH5 knockdown and LINC00968 overexpression on global m6A levels. **(D)** Cell viability of RA-PMNs was assessed using CCK-8 assays under ALKBH5 silencing and LINC00968 overexpression. **(E, F)** Flow cytometry was performed to analyze cell cycle distribution in RA-PMNs after ALKBH5 knockdown and LINC00968 overexpression. **(G)** mRNA expression of DNase I was determined by RT-qPCR following ALKBH5 silencing and LINC00968 overexpression. **(H)** NE expression levels were quantified via ELISA after ALKBH5 knockdown and LINC00968 overexpression. **(I, J)** Immunofluorescence (IF) staining was used to evaluate the expression and localization of NE (green) and MPO (red) in RA-PMNs under the indicated treatments. All data are presented as mean ± SD. *p < 0.05, **p < 0.01, ***p < 0.001.

To further verify whether LINC00968 is the key mediator through which ALKBH5 regulates neutrophil survival, cell cycle delay, and NET formation, we overexpressed LINC00968 in RA-PMNs with ALKBH5 knockdown. As expected, overexpression of LINC00968 not only significantly increased LINC00968 expression levels but also reduced the total m6A level and the m6A modification level of LINC00968 in RA-PMNs. Moreover, LINC00968 overexpression effectively rescued the effects of ALKBH5 knockdown on the gene expression and m6A modification levels of LINC00968, as well as the global m6A level (**Figures 5A–C**). CCK-8 assays showed that LINC00968 overexpression markedly promoted RA-PMN proliferation and reversed the suppressive effect of ALKBH5 silencing on cell viability (**Figure 5D**). Flow cytometry analysis revealed that, compared with the OE-NC group, LINC00968 overexpression significantly increased the proportion of RA-PMNs in the G1 phase and decreased the proportion in the G2 phase. Furthermore, overexpression of LINC00968 in ALKBH5-knockdown cells rescued the distribution of cell cycle phases (G1 and G2) in RA-PMNs (**Figures 5E, F**). Subsequent RT-qPCR results confirmed that LINC00968 overexpression suppressed DNase I mRNA expression and enhanced NE expression, thereby counteracting the increased DNase I and decreased NE levels induced by ALKBH5 knockdown (**Figures 5G, H**). Additionally, immunofluorescence staining further demonstrated that under conditions of ALKBH5 knockdown and LINC00968 overexpression, the expression and nuclear localization of NE and MPO in RA-PMNs were significantly reduced (**Figures 5I, J**). These results indicate that ALKBH5 regulates neutrophil proliferation, cell cycle progression, and NETs formation by mediating the m6A modification of LINC00968.

### 3.6 ALKBH5 regulates oxidative stress-inflammatory response in RA-PMNs by mediating m6A modification of LINC00968

**Figure 6:**
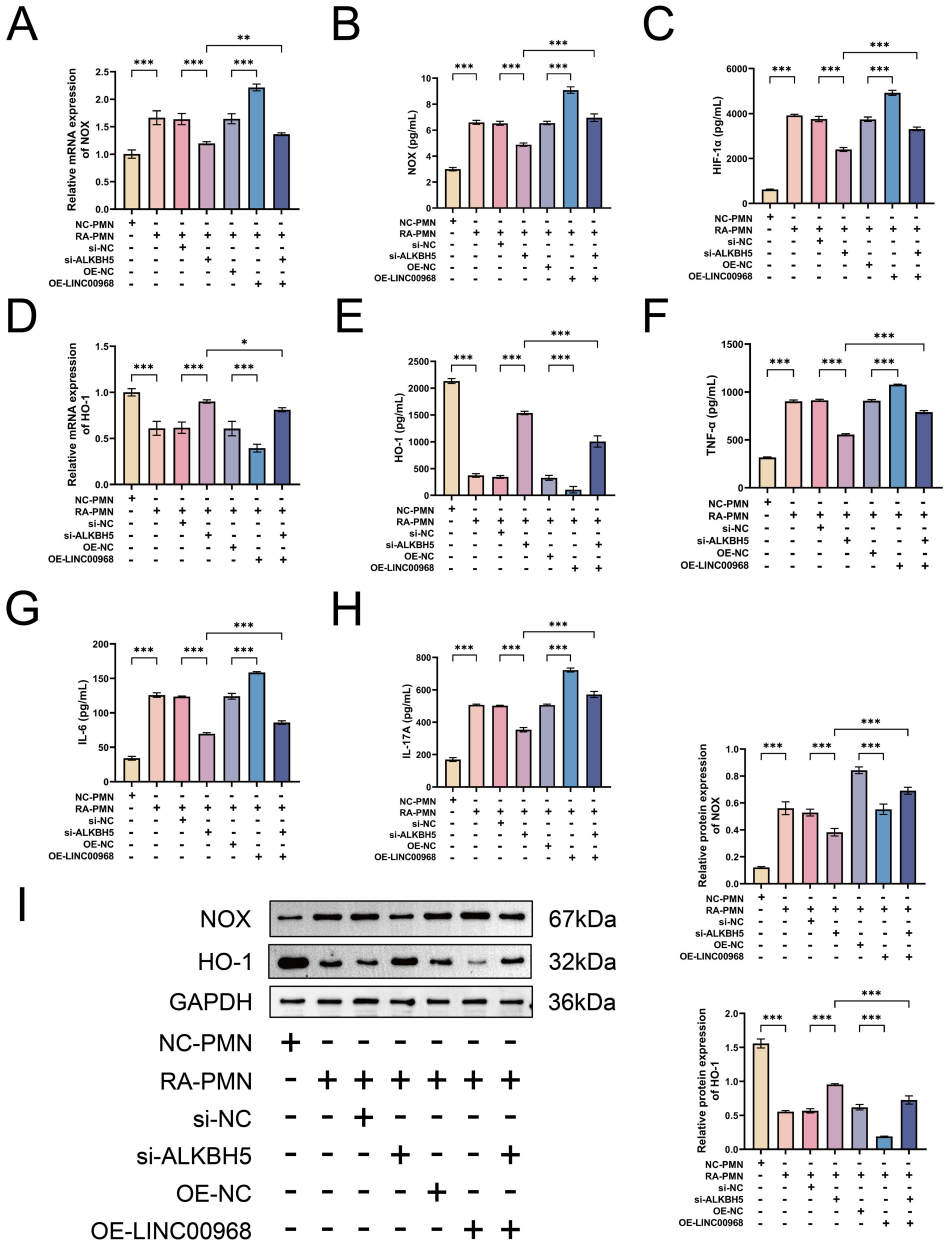
ALKBH5 regulates oxidative stress-inflammatory response in RA-PMNs via m6A modification of LINC00968. **(A)** NOX mRNA levels after ALKBH5 knockdown and LINC00968 overexpression were detected by RT-qPCR. **(B)** NOX protein levels were measured by ELISA. **(C)** HIF-1a mRNA levels were assessed via RT-qPCR. **(D)** HO-1 mRNA levels were determined by RT-qPCR. **(F)** HO-1 protein levels were quantified using ELISA. **(F–H)** TNF-a, IL-6, and IL-17A levels were evaluated by ELISA. **(I)** Protein expression of NOX and HO-1 was examined by Western blot. Data are presented as mean ± SD. *p < 0.05, **p < 0.01, ***p < 0.001.

We further investigated whether LINC00968 plays a key role in ALKBH5-mediated regulation of oxidative stress and inflammation in RA-PMNs. Our data showed that overexpression of LINC00968 significantly increased NOX expression and rescued the downregulation of NOX induced by ALKBH5 knockdown (**Figures 6A, B**). RT-qPCR results also demonstrated that LINC00968 overexpression markedly upregulated HIF-1α mRNA levels compared with the NC group, whereas this effect was reversed when ALKBH5 was silenced (**Figure 6C**). Subsequent analysis of HO-1 revealed that LINC00968 overexpression elevated HO-1 levels and counteracted the increase in HO-1 induced by ALKBH5 knockdown (**Figures 6D, E**). Furthermore, ELISA assays showed that LINC00968 overexpression enhanced the secretion of TNF-α, IL-6, and IL-17A, and these inflammatory cytokines were further upregulated when LINC00968 was overexpressed under ALKBH5-silenced conditions (**Figures 6F–H**). Western blot results further confirmed the effects of ALKBH5 knockdown and LINC00968 overexpression on NOX and HO-1 protein expression (**Figure 6I**). These results indicate that ALKBH5 modulates oxidative stress and inflammatory responses in RA-PMNs through m6A-dependent regulation of LINC00968.

### 3.7 XFC inhibits cocultured RA-PMN abnormal survival and NETosis by reversing ALKBH5-mediated m6A modification of LINC00968

**Figure 7:**
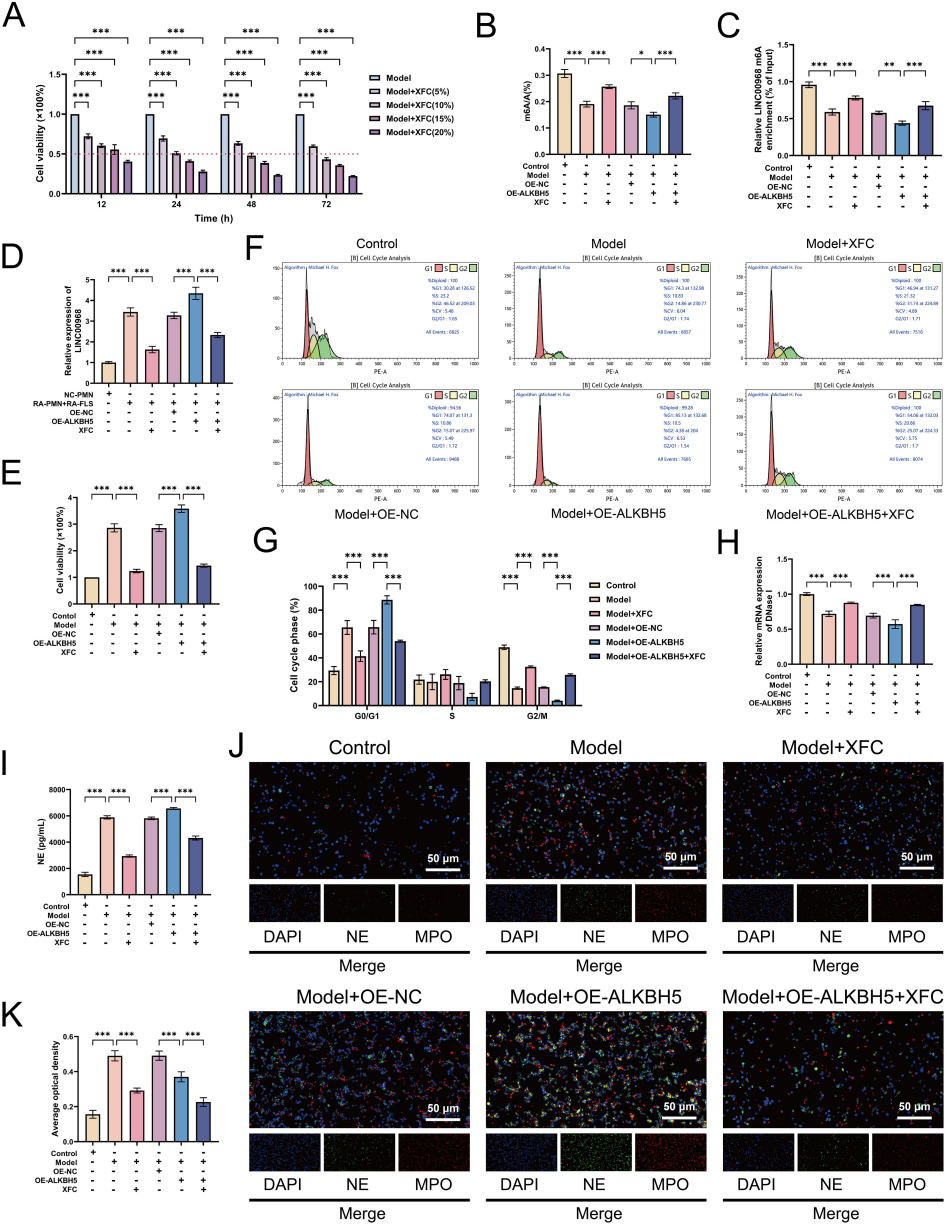
XFC inhibits proliferation, cell cycle arrest, and NETosis in cocultured RA-PMNs by reversing ALKBH5-mediated m6A modification of LINC00968. **(A)** CCK-8 assay evaluating effects of different XFC concentrations on cell proliferation at 12, 24, 48, and 72 (h) **(B)** Global m6A levels after XFC treatment and/or ALKBH5 overexpression. **(C)** m6A levels of LINC00968 detected by MeRIP-qPCR. **(D)** LINC00968 expression measured by RT-qPCR. **(E)** RA-PMN viability assessed by CCK-8. **(F, G)** Cell cycle distribution analyzed by flow cytometry. **(H)** DNase I mRNA levels determined via RT-qPCR. **(I)** NE protein levels measured by ELISA. **(J, K)** Immunofluorescence staining of NE (green) and MPO (red). Data are presented as mean ± SD. *p < 0.05, **p < 0.01, ***p < 0.001.

To elucidate the mechanism of XFC in the complex pathological environment of RA, we established an *in vitro* coculture system involving RA-PMNs and RA-FLS. Previous studies indicated that a RA-PMN: RA-FLS ratio of 3:1 at 48 h yielded the most pronounced proliferation and inflammatory response in RA-FLSs; subsequent experiments were conducted under these conditions (29). CCK-8 assays showed that XFC-containing serum reduced RA-FLS proliferation in a time- and concentration-dependent manner. A 10% XFC serum concentration, which inhibited cell viability by 50% at 24 h, was selected for further experiments (**Figure 7A**). Compared with the model group, XFC treatment increased global m6A levels and m6A modification of LINC00968, whereas reducing LINC00968 expression. Overexpression of ALKBH5 reversed these effects (**Figures 7B–D**). CCK-8 assays indicated that XFC suppressed RA-PMN proliferation and attenuated the pro-proliferative effect of ALKBH5 overexpression (**Figure 7E**). Flow cytometry revealed that XFC decreased the proportion of cells in the G1 phase and increased those in the G2 phase. These effects were reversed upon ALKBH5 overexpression (**Figures 7F, G**). Additionally, XFC increased DNase I mRNA and decreased NE expression, counteracting the effects of ALKBH5 overexpression (**Figures 7H, I**). Immunofluorescence staining confirmed that XFC reduced nuclear localization of NE and MPO, an effect that was enhanced under ALKBH5-overexpressing conditions (**Figures 7J, K**). These results demonstrate that XFC suppresses RA-PMN proliferation, cell cycle arrest, and NETosis by reversing ALKBH5-mediated m6A modification of LINC00968.

### 3.8 XFC reverses oxidative stress-inflammatory imbalance by downregulating ALKBH5 expression

**Figure 8:**
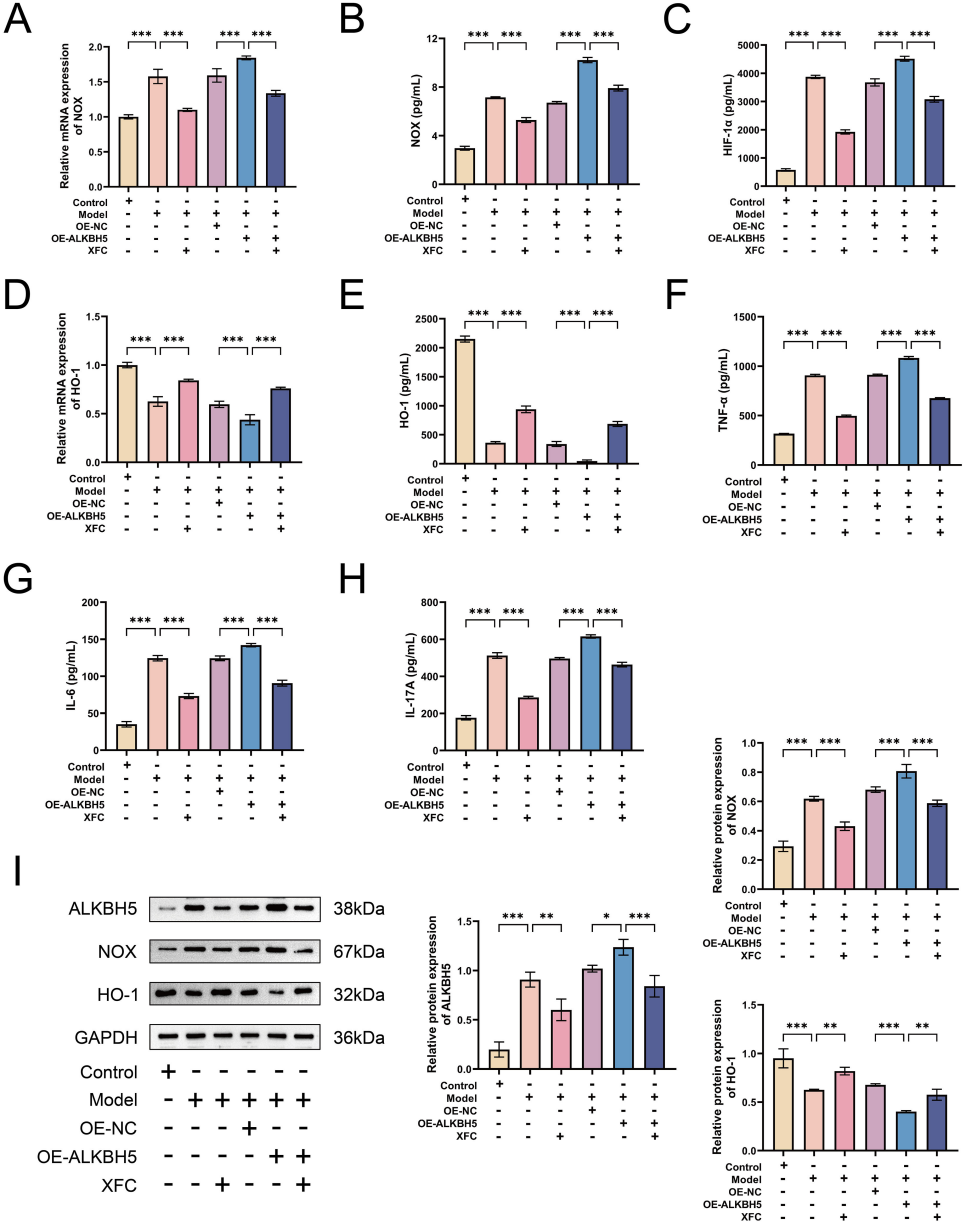
XFC reverses oxidative stress-inflammatory dysregulation by downregulating ALKBH5. **(A)** NOX mRNA levels after XFC treatment and/or ALKBH5 overexpression. **(B)** NOX protein levels measured by ELISA. **(C)** HIF-1α mRNA levels via RT-qPCR. **(D)** HO-1 mRNA levels by RT-qPCR. **(E)** HO-1 protein levels by ELISA. **(F–H)** TNF-α, IL-6, and IL-17A levels by ELISA. **(I)** Protein levels of ALKBH5, NOX, and HO-1 by Western blot. Data are presented as mean ± SD. *p < 0.05, **p < 0.01, ***p < 0.001.

We further explored the effect of XFC on oxidative stress and inflammation. XFC treatment reduced NOX expression and reversed the upregulation of NOX induced by ALKBH5 overexpression (**Figures 8A, B**). RT-qPCR showed that XFC downregulated HIF-1α mRNA, and this effect was reversed when ALKBH5 was overexpressed (**Figure 8C**). XFC also increased the HO-1 expression and counteracted the reduction in HO-1 caused by ALKBH5 overexpression (**Figures 8D, E**). ELISA results confirmed that XFC reduced TNF-α, IL-6, and IL-17A levels, and these cytokines were further suppressed when XFC was combined with ALKBH5 overexpression (**Figures 8F–H**). Western blot analysis demonstrated that XFC downregulated ALKBH5 and reversed the effects of ALKBH5 overexpression on NOX and HO-1 (**Figure 8I**). These findings indicate that XFC ameliorates oxidative stress and inflammatory responses by inhibiting ALKBH5 expression.

### 3.9 XFC acts through the ALKBH5–m6A–LINC00968 pathway to attenuate oxidative stress and NETosis in RA

**Figure 9:**
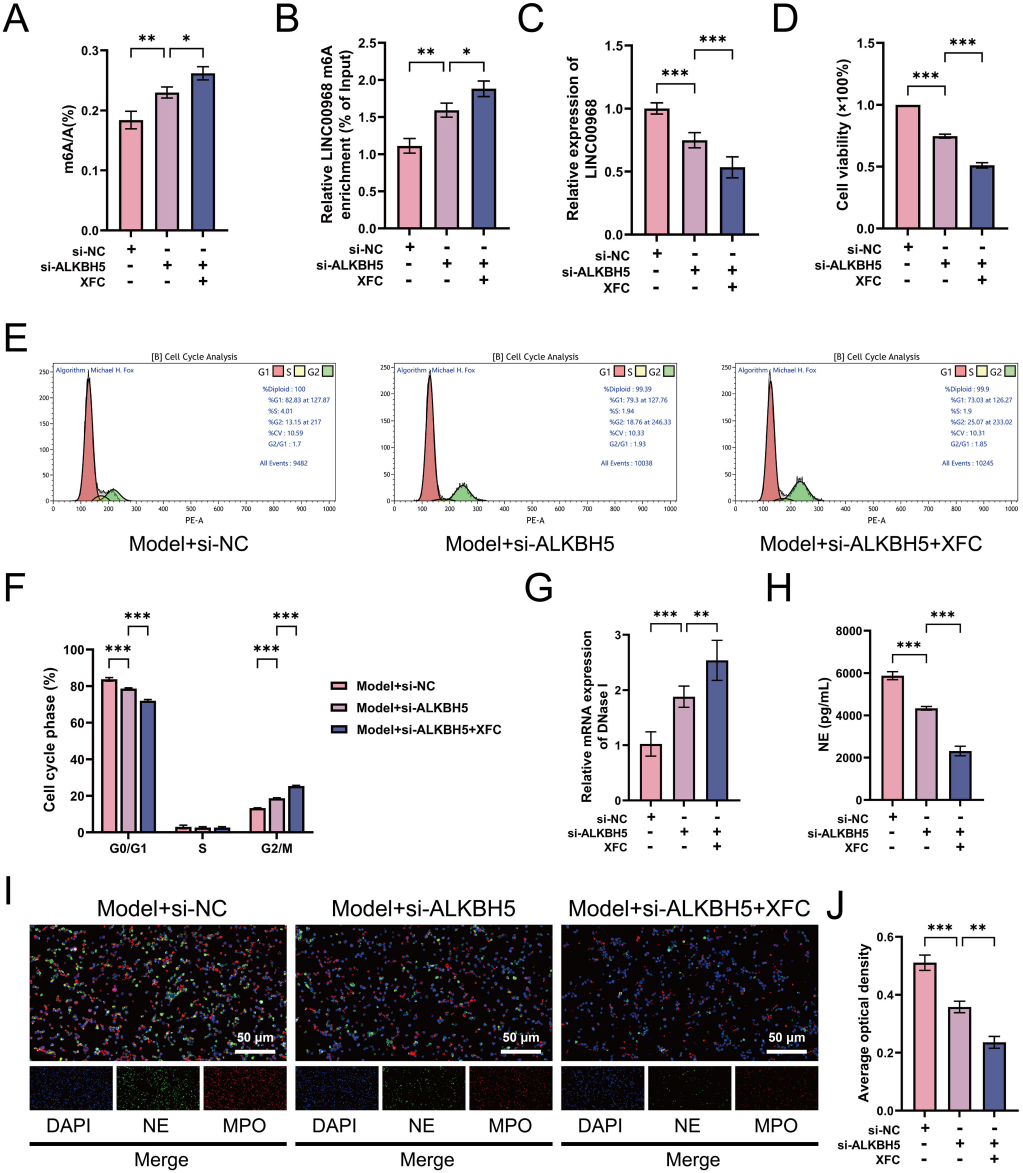
XFC attenuates RA-PMN hyperactivation and NETosis by modulating the ALKBH5/LINC00968/m6A axis. **(B)** LINC00968 m6A levels by MeRIP-qPCR. **(C)** LINC00968 expression by RT-qPCR. **(D)** Cell viability by CCK-8. **(E, F)** Cell cycle analysis by flow cytometry. **(G)** DNase I mRNA by RT-qPCR. **(H)** NE protein by ELISA. **(I, J)** Immunofluorescence of NE (green) and MPO (red). Data are presented as mean ± SD. *p < 0.05, **p < 0.01, ***p < 0.001.

**Figure 10:**
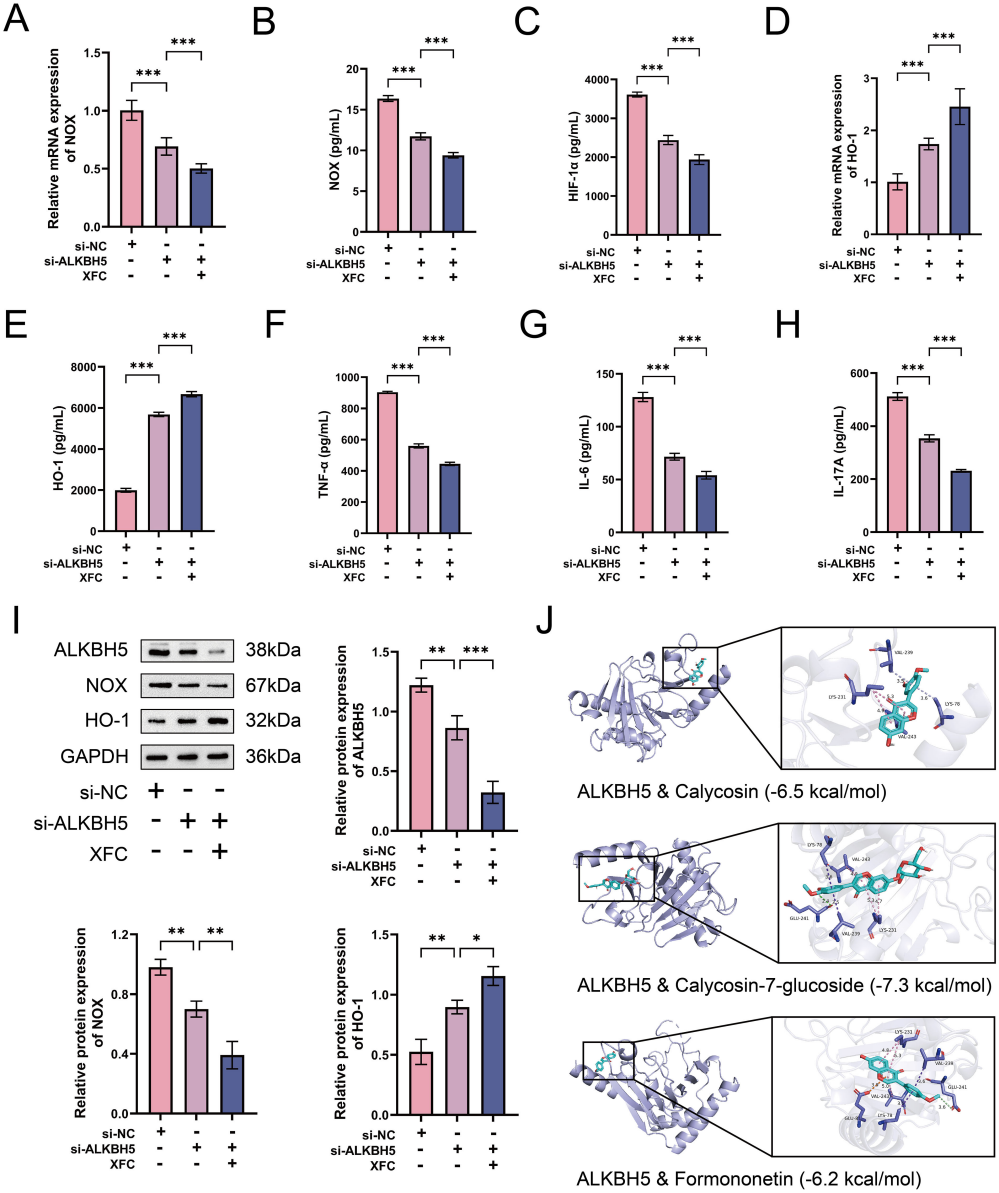
XFC alleviates oxidative stress and inflammatory responses through regulation of ALKBH5. **(A)** NOX mRNA after ALKBH5 knockdown and XFC treatment. **(B)** NOX protein by ELISA. **(C)** HIF-1α mRNA by RT-qPCR. **(D)** HO-1 mRNA by RT-qPCR. **(E)** HO-1 protein by ELISA. **(F–H)** TNF-α, IL-6, and IL-17A levels by ELISA. **(I)** Protein levels of ALKBH5, NOX, and HO-1 by Western blot. **(J)** Molecular docking of calycosin, calycosin-7-glucoside, and formononetin with ALKBH5. Data are presented as mean ± SD. *p < 0.05, **p < 0.01, ***p < 0.001.

To determine whether XFC acts through ALKBH5-mediated m6A modification of LINC00968, we treated ALKBH5-silenced cells with XFC. As expected, XFC further increased global m6A levels and m6A modification of LINC00968, while reducing LINC00968 expression (**Figures 9A–C**). CCK-8 and flow cytometry assays showed that XFC enhanced the reduction in cell viability and promoted cell cycle recovery induced by ALKBH5 knockdown (**Figures 9D–F**). Similarly, XFC increased DNase I mRNA and decreased NE expression (**Figures 9G, H**). Immunofluorescence staining confirmed that XFC further reduced nuclear NE and MPO staining in ALKBH5-silenced cells (**Figures 9I, J**). Moreover, XFC inhibited NOX and HIF-1α expression and promoted HO-1 expression under ALKBH5-silenced conditions (**Figures 10A–E**). ELISA showed that XFC further suppressed TNF-α, IL-6, and IL-17A levels in ALKBH5-knockdown cells (**Figures 10F–H**). Western blot results supported these observations (**Figure 10I**). Previous HPLC fingerprinting identified three active components in XFC: calycosin, calycosin-7-glucoside, and formononetin (23). Molecular docking simulations indicated that these compounds exhibit strong binding affinity to ALKBH5 (**Figure 10J**). These results suggest that XFC exerts its therapeutic effects in RA by functionally modulating the ALKBH5–m6A–LINC00968 pathway, ultimately curbing neutrophil dysregulation and the resultant inflammatory damage.

### 3.10 XFC suppresses RA-PMN hyperactivation and NETosis via the ALKBH5–m6A–LINC00968 axis to attenuate oxidative stress-inflammatory responses

**Figure 11:**
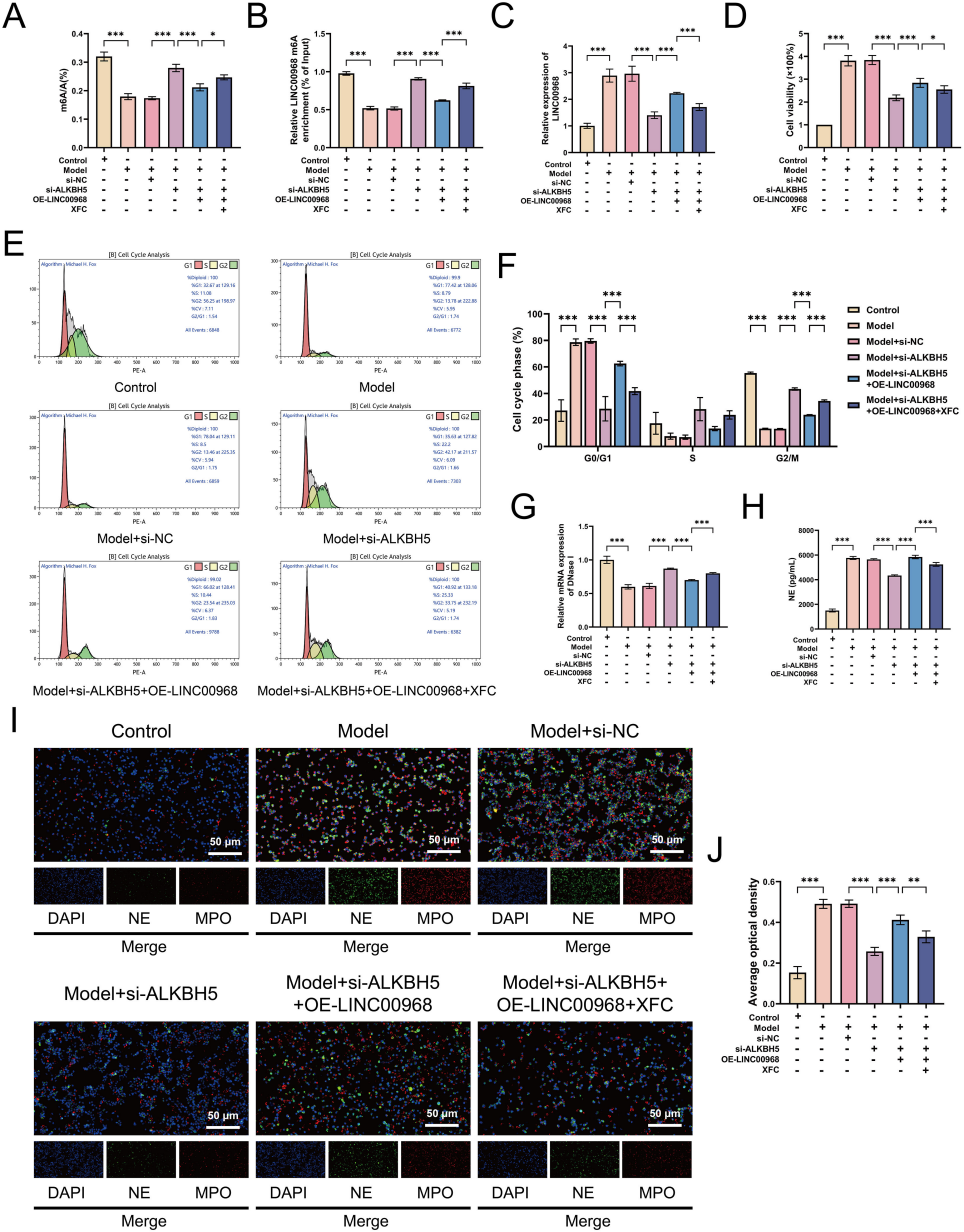
XFC suppresses cocultured RA-PMN hyperactivation and NETosis via the ALKBH5–m6A–LINC00968 axis. **(A)** Global m6A levels after ALKBH5 knockdown, LINC00968 overexpression, and XFC treatment. **(B)** LINC00968 m6A levels by MeRIP-qPCR. **(C)** LINC00968 expression by RT-qPCR. **(D)** Cell viability by CCK-8. **(E, F)** Cell cycle analysis by flow cytometry. **(G)** DNase I mRNA by RT-qPCR. **(H)** NE protein by ELISA. **(I, J)** Immunofluorescence of NE (green) and MPO (red). Data are presented as mean ± SD. *p < 0.05, **p < 0.01, ***p < 0.001.

**Figure 12:**
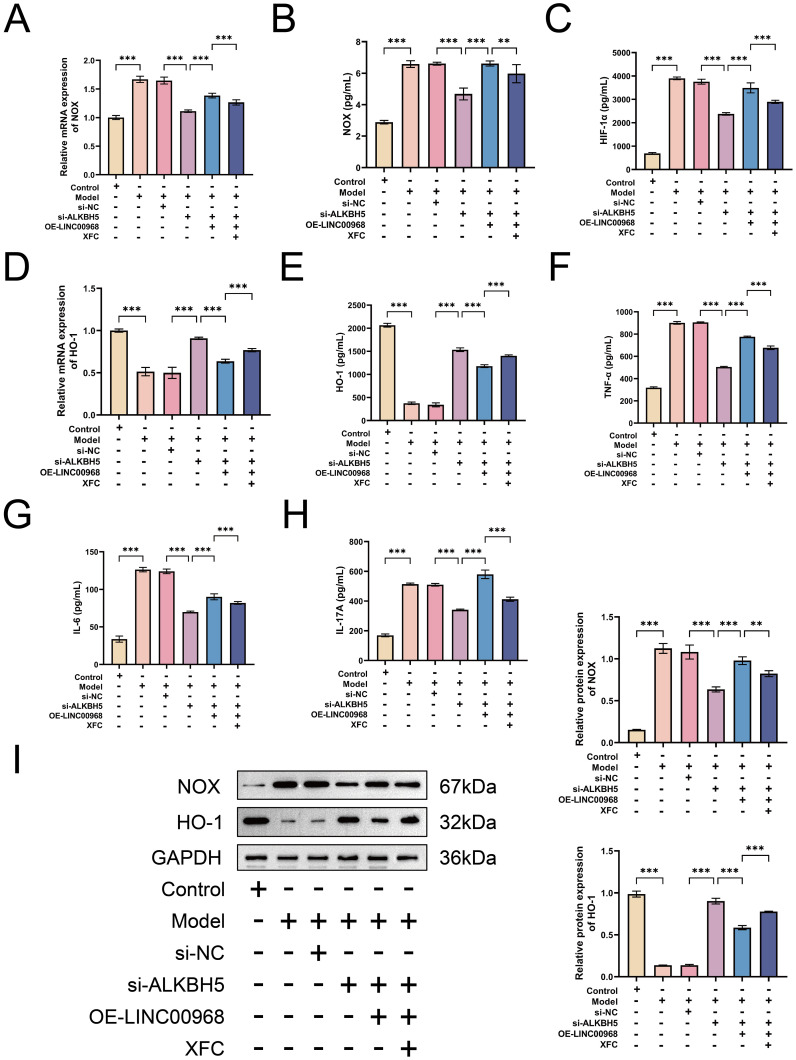
The ALKBH5–m6A–LINC00968 axis mediates the protective effect of XFC against oxidative stress and inflammation. **(A)** NOX mRNA after ALKBH5 knockdown, LINC00968 overexpression, and XFC treatment. **(B)** NOX protein by ELISA. **(C)** HIF-1α mRNA by RT-qPCR. **(D)** HO-1 mRNA by RT-qPCR. **(E)** HO-1 protein by ELISA. **(F–H)** TNF-α, IL-6, and IL-17A levels by ELISA. **(I)** Protein levels of ALKBH5, NOX, and HO-1 by Western blot. Data are presented as mean ± SD. **p < 0.01, ***p < 0.001.

To determine whether LINC00968 is involved in the mechanism through which XFC modulates ALKBH5 activity, we overexpressed LINC00968 in ALKBH5-silenced cells and treated them with XFC. XFC reversed the effects of LINC00968 overexpression by increasing global m6A levels and m6A modification of LINC00968 and decreasing LINC00968 expression (**Figures 11A–C**). CCK-8 and flow cytometry showed that XFC inhibited RA-PMN proliferation and promoted cell cycle recovery even when LINC00968 was overexpressed (**Figures 11D–F**). Similarly, XFC reversed the decrease in DNase I mRNA, increase in NE expression, and enhanced nuclear NE and MPO staining caused by LINC00968 overexpression (**Figures 11G–J**). Furthermore, XFC inhibited NOX and HIF-1α expression and promoted HO-1 expression under these conditions (**Figures 12A–E**). ELISA confirmed that XFC suppressed the upregulation of TNF-α, IL-6, and IL-17A induced by LINC00968 overexpression (**Figures 12F–H**). Western blot analysis further supported these findings (**Figure 12I**). These results demonstrate that XFC upregulates m6A modification of LINC00968 via ALKBH5, thereby inhibiting RA-PMN hyperactivation, NETosis, and oxidative stress-inflammatory cascades.

## 4 Discussion

RA is a refractory autoimmune disease characterized by multifactor-induced immune-inflammatory responses. In recent years, the role of long non-coding RNAs (lncRNAs) and their methylated modifications has been extensively investigated in the pathological mechanisms of RA (30). LncRNAs can participate in the assembly of histone modification complexes, promote the activation of immune signaling pathways, engage in posttranscriptional processes such as mRNA splicing, directly interact with mRNAs via homologous regions leading to mRNA degradation, and function as competing endogenous RNAs (ceRNAs) by sequestering miRNAs (31). Among various RNA modifications, N6-methyladenosine (m6A) is the most prevalent posttranscriptional modification in eukaryotic messenger RNAs (mRNAs) and non-coding RNAs (ncRNAs) (32). Due to its crucial roles in RNA splicing, export, stability, and translation, m6A modification has emerged as a highly promising research field and predictive value of medication efficacy.

The design of this study was grounded in a solid clinical foundation. Our retrospective analysis of a large RA cases initially confirmed that XFC is associated with multidimensional immunological and inflammatory amelioration, including improvements in novel composite inflammatory markers, without inducing hepatic or renal impairment. These composite indicators, including NLR, MLR, and SII, have in recent years been demonstrated to provide a more comprehensive reflection of systemic inflammatory states and immune dysregulation in RA patients, with their diagnostic value increasingly recognized (33–35). After establishing its efficacy through conventional statistics, we further employed data mining and dynamic modeling to deconstruct this complex clinical phenotype. In association rule analysis, although the derived lift values approached 1.0, this precisely provided crucial insight: It indicated not weak associations, but rather the pervasive presence of clinical improvement. Indeed, patients in the XFC group exhibited improvements across multiple inflammatory markers post-treatment, rendering parameter enhancement a universal outcome and consequently diluting the lift value of any specific rule. Similarly, a previous cohort study involving 10,000 participants revealed a robust and long-term protective association between XFC usage and reduced readmission rates among RA patients (20). Thus, the core insight of this analysis lies in revealing the systemic, multidimensional anti-inflammatory pattern exhibited by XFC as an adjunct therapeutic strategy. This clinical feature, coupled with the cumulative efficacy revealed by the random walk model, jointly guided subsequent mechanistic hypotheses, prompting us to explore upstream regulatory mechanisms capable of coordinating such extensive inflammatory resolution.

Previous research and bioinformatic analyses had indicated that neutrophil dysfunction and epitranscriptomic regulation play critical roles in RA pathogenesis (16). Transmission electron microscopy revealed impaired neutrophil membranes in RA patients alongside characteristic NET structures (36). Guided by these findings, we shifted our focus from broadly exploring the potential mechanisms to rigorously validating this specific, hypothesis-driven pathway. The ensuing experiments were designed to mechanistically link the ALKBH5/LINC00968/m^6^A axis to the systemic clinical benefits of XFC observed in our study. We initially used the online tool SRAMP to predict three potential m6A modification sites on LINC00968. Subsequent experiments showed that ALKBH5 expression was significantly upregulated in RA neutrophils, leading to reduced global m6A levels and altered RNA stability and translation efficiency. Therefore, we selected ALKBH5 as the main regulator of LINC00968 m6A modification involved in RA disease activity and immune-inflammatory responses, suggesting LINC00968 as a potential m6A target of ALKBH5. Functional experiments confirmed that overexpression of ALKBH5 or LINC00968 enhances NETosis and inflammatory responses, whereas silencing these molecules significantly suppresses these effects. These results underscore the importance of the ALKBH5–LINC00968–m6A axis in regulating neutrophil activation, indicating that this pathway is a critical epigenetic switch in RA pathogenesis. However, the precise mechanisms by which this methylation modification modulates NETosis remain to be fully elucidated. NETs are a specialized defense mechanism of neutrophils and also a source of citrullinated autoantigens and inflammatory factors that can activate RA-FLS, leading to joint damage (37, 38). NETs produced by neutrophils in RA synovium can selectively stimulate the expression of pro-inflammatory genes in synoviocytes, suggesting that NETosis plays an essential role in RA immune inflammation under oxidative stress (6). Dysregulation of the ALKBH5/LINC00968 axis profoundly affects neutrophil dysfunction in RA. We observed that ALKBH5 overexpression and consequent LINC00968 hypomethylation induce significant alterations in cell cycle distribution, manifesting as a highly active terminal differentiation state rather than proliferative arrest. Consistent with this, neutrophils exhibited enhanced metabolic activity (elevated CCK-8 values), providing the necessary bioenergetic and biosynthetic support for NETosis. Enhanced oxidative stress and mitochondrial dysfunction further promote pro-inflammatory cytokine release and NET formation. These phenotypic changes are intrinsically linked through metabolic reprogramming, supplying both substrates and signals for sustained neutrophil activation and NETosis.

Previous genetic association studies in Chinese populations have revealed that genetic variations in NADPH oxidase genes are linked to RA susceptibility and specific clinical features, with rs4821544 and rs729749 polymorphisms potentially associated with RA risk, further supporting the involvement of the NADPH pathway in RA pathogenesis (39). However, the mechanism by which this pathway contributes to erosive arthritis in RA remains unclear. Dysregulation of the ALKBH5/LINC00968 axis significantly exacerbates neutrophil dysfunction in RA through the NADPH pathway. We found that ALKBH5 overexpression and subsequent LINC00968 hypomethylation catalyzes the conversion of NADP^+^ to NADPH, leading to intracellular NADPH accumulation. On one hand, high NADPH levels provide sufficient reducing power for glutathione (GSH) synthesis, temporarily enhancing the capacity to neutralize ROS, which feedback induces mitochondria to produce more ROS, resulting in an “oxidative burst”. On the other hand, NOX is activated, further catalyzing the conversion of molecular oxygen into superoxide, collectively causing a sharp increase in oxidative stress (40). This intense oxidative stress not only triggers NETosis but also directly leads to mitochondrial membrane depolarization, respiratory chain dysfunction, and aberrant energy metabolism (41). Metabolic reprogramming (e.g., increased CCK-8 activity) provides bioenergetic and substrate support for NETosis, whereas dysregulated NADPH metabolism amplifies inflammatory factor release and autoantigen exposure by promoting inflammasome activation and histone citrullination, ultimately forming a positive feedback loop that perpetuates immune-inflammatory responses (42).

**Figure 13:**
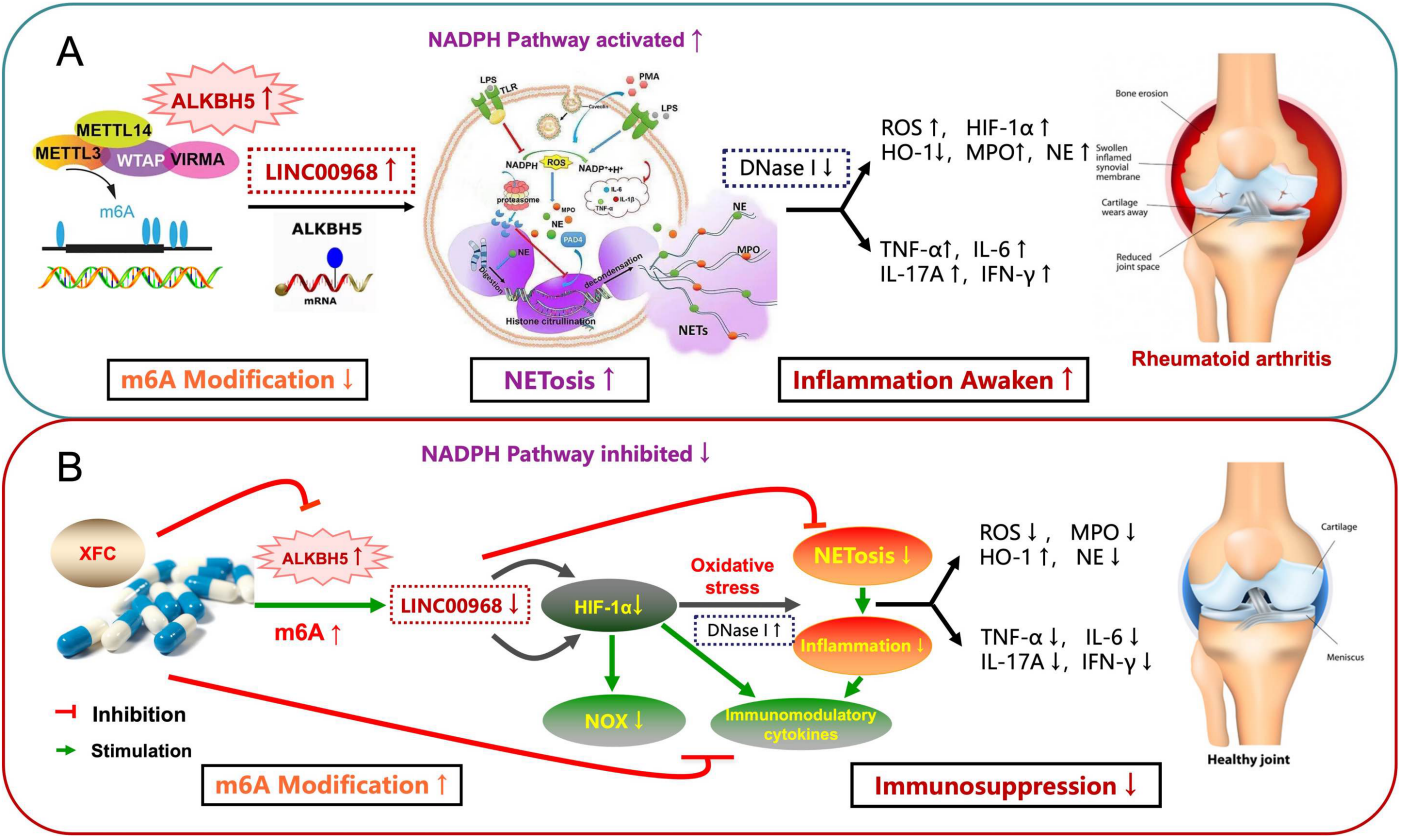
Schematic diagram summarizing the main findings of the present study.

Growing evidence confirms that Chinese herbal medicines can modulate immune-inflammatory responses in RA by targeting inflammation mechanisms. For instance, the TCM formula Wangbi Granules has been shown to alleviate RA by inhibiting FAPα expression and regulating AKT/mTOR pathway phosphorylation (43). Based on previous research, we conclude that the pathogenesis of RA is characterized by “spleen deficiency with dampness excess and phlegm-stasis obstruction”, which aligns with immune-inflammatory responses and oxidative stress imbalance in RA. Sun et al. demonstrated that XFC can upregulate LINC00638 expression and activate the Nrf2/HO-1 pathway, thereby suppressing inflammation and oxidative stress in RA (21). Previous pharmacological studies have also revealed that XFC inhibits FTO, thereby increasing m6A levels in ENST00000619282 and suppressing its evasion of apoptosis in cocultured RA-FLS (44). Our cellular and functional experiments consistently demonstrate that XFC treatment leads to the inhibition of ALKBH5 function, which in turn enhances the m6A methylation of LINC00968 and suppresses the subsequent oxidative stress and NETosis cascade. To explore a potential mechanism for this regulation, we performed molecular docking simulations. The results predicted that several bioactive components of XFC could potentially interact with ALKBH5, suggesting one plausible mechanism for the observed functional regulation. However, future studies are required to experimentally validate this putative interaction. Thus, XFC exerts therapeutic effects via the ALKBH5/LINC00968/m6A pathway, providing a compelling example of TCM targeting epitranscriptomic mechanisms in autoimmune diseases (**Figure 13**).

A significant strength of this study is the first demonstration of the critical role of m6A modification in regulating NETosis in RA neutrophils, offering new epitranscriptomic insights and potential therapeutic targets for RA. However, several limitations should be acknowledged. First, although propensity score matching was applied to improve comparability between the XFC and non-XFC groups, the inherent limitations of a retrospective design remain. Nevertheless, the consistent positive signals across multiple immune-inflammatory parameters, validated by dynamic modeling, provide strong clinical support for the observed associations and fully justify subsequent mechanistic investigation. Future prospective multicenter cohort studies will be conducted to definitively establish the causal therapeutic effects of XFC and to validate the relationships identified in this retrospective analysis. Second, the primary conclusions are largely derived from *in vitro* experiments. Moreover, the use of drug-containing serum prepared from SD rats to treat human RA neutrophils introduces limitations associated with interspecies differences in drug metabolism and component biotransformation. Future work will include validation in animal models. Third, although cellular experiments strongly support the functional role of the ALKBH5/LINC00968/m^6^A axis, the molecular docking predictions require experimental confirmation through techniques such as surface plasmon resonance or cellular thermal shift assays to directly verify the physical interaction between XFC components and ALKBH5. Finally, although LINC00968 was identified as a key mediator of ALKBH5-dependent m^6^A modification, its precise molecular mechanisms, including specific binding partners and downstream effectors, remain incompletely elucidated. Future studies will employ high-throughput approaches such as RNA pull-down and RIP-qPCR to systematically characterize its functional network.

## 5 Conclusion

In summary, our study unveils a critical role of ALKBH5-mediated m6A demethylation of LINC00968 in promoting oxidative stress and NETosis in RA neutrophils via the NADPH pathway. These findings provide novel insights into the epitranscriptomic regulation of neutrophil activation and highlight the ALKBH5–LINC00968–m6A axis as a potential therapeutic target in RA. Moreover, we demonstrate that the traditional Chinese medicine formulation XFC exerts protective effects by modulating this pathway, thereby suppressing NETosis and mitigating inflammatory responses. This work not only advances our understanding of m6A methylation in autoimmune diseases but also supports the future development of targeted interventions leveraging traditional medicine and epitranscriptomic mechanisms.

## Data Availability

The original contributions presented in the study are included in the article/**Supplementary Material**. Further inquiries can be directed to the corresponding author.

## References

[1] Reis LR Nascimento RO Massafera MP Di Mascio P Ronsein GE . Investigating neutrophil responses to stimuli: Comparative analysis of reactive species-dependent and independent mechanisms. Redox Biol. (2025) 81:103540. doi: 10.1016/j.redox.2025.103540, PMID: 40037225 PMC11923813

[2] Tang J Xia J Gao H Jiang R Xiao L Sheng H . IL33-induced neutrophil extracellular traps (NETs) mediate a positive feedback loop for synovial inflammation and NET amplification in rheumatoid arthritis. Exp Mol Med. (2024) 56:2602–16. doi: 10.1038/s12276-024-01351-7, PMID: 39617790 PMC11671579

[3] Bertrand D De Meyst E Doumen M De Cock D Joly J Neerinckx B . Effectiveness and patient-reported impact of on-flare retreatment in patients with rheumatoid arthritis: insights from retrospective long-term real-world data. BMC Rheumatol. (2025) 9:91. doi: 10.1186/s41927-025-00530-x, PMID: 40691804 PMC12278676

[4] Song YJ Cho SK Choi SR Lee SS Lee HS Park SH . Factors influencing delayed attainment of low disease activity in rheumatoid arthritis patients continuing targeted therapy. Korean J Intern Med. (2025) 40:835–44. doi: 10.3904/kjim.2024.344, PMID: 40916340 PMC12425689

[5] Adami G Fassio A Rossini M Bertelle D Pistillo F Benini C . Tapering glucocorticoids and risk of flare in rheumatoid arthritis on biological disease-modifying antirheumatic drugs (bDMARDs). RMD Open. (2023) 9:e002792. doi: 10.1136/rmdopen-2022-002792, PMID: 36599630 PMC9815081

[6] Ye H Wang H Han B Chen K Wang X Ma F . Guizhi Shaoyao Zhimu decoction inhibits neutrophil extracellular traps formation to relieve rheumatoid arthritis via gut microbial outer membrane vesicles. Phytomedicine. (2025) 136:156254. doi: 10.1016/j.phymed.2024.156254, PMID: 39586125

[7] Wu S Peng W Liang X Wang W . Anti-citrullinated protein antibodies are associated with neutrophil extracellular trap formation in rheumatoid arthritis. J Clin Lab Anal. (2021) 35:e23662. doi: 10.1002/jcla.23662, PMID: 33249645 PMC7957993

[8] Hu B Li D Zeng Z Zhang Z Cao R Dong X . Integrated proteome and malonylome analyses reveal the neutrophil extracellular trap formation pathway in rheumatoid arthritis. J Proteomics. (2022) 262:104597. doi: 10.1016/j.jprot.2022.104597, PMID: 35489682

[9] Jiang H Lu Q Xu J Huo G Cai Y Geng S . Sinomenine ameliorates adjuvant-induced arthritis by inhibiting the autophagy/NETosis/inflammation axis. Sci Rep. (2023) 13:3933. doi: 10.1038/s41598-023-30922-3, PMID: 36894604 PMC9998614

[10] Huang SU O'Sullivan KM . The expanding role of extracellular traps in inflammation and autoimmunity: the new players in casting dark webs. Int J Mol Sci. (2022) 23:3793. doi: 10.3390/ijms23073793, PMID: 35409152 PMC8998317

[11] Cross AL Wright HL Choi J Edwards SW Ruiz-Opazo N Herrera VLM . Circulating neutrophil extracellular trap-forming neutrophils in rheumatoid arthritis exacerbation are majority dual endothelin-1/signal peptide receptor+ subtype. Clin Exp Immunol. (2024) 218:163–8. doi: 10.1093/cei/uxae072, PMID: 39110036 PMC11482496

[12] O'Neil LJ Oliveira CB Wang X Navarrete M Barrera-Vargas A Merayo-Chalico J . Neutrophil extracellular trap-associated carbamylation and histones trigger osteoclast formation in rheumatoid arthritis. Ann Rheum Dis. (2023) 82:630–8. doi: 10.1136/ard-2022-223568, PMID: 36737106 PMC11302494

[13] Wen J Liu J Wan L Sun Y Wang F . Crosstalk between N6-methyladenosine modification and ncRNAs in rheumatic diseases: therapeutic and diagnostic implications. Inflammation Res. (2025) 74:79. doi: 10.1007/s00011-025-02034-3, PMID: 40402257

[14] Zhao SS Yuan C Liu JL Wu QC Zhou XL . SETD2 drives METTL14-mediated m6A to suppress Piezo1 Attenuation and activate TGM2 to promote pulmonary hypertension. Cell Mol Life Sci. (2025) 82:302. doi: 10.1007/s00018-025-05809-3, PMID: 40778995 PMC12334401

[15] Fan D Geng Q Wang B Wang X Xia Y Yang L . Hypoxia-induced ALKBH5 aggravates synovial aggression and inflammation in rheumatoid arthritis by regulating the m6A modification of CH25H. Clin Immunol. (2024) 261:109929. doi: 10.1016/j.clim.2024.109929, PMID: 38331303

[16] Li Y Liu J Sun Y Hu Y Zhou Q Cong C . Deciphering hub genes and immune landscapes related to neutrophil extracellular traps in rheumatoid arthritis: insights from integrated bioinformatics analyses and experiments. Front Immunol. (2024) 15:1521634. doi: 10.3389/fimmu.2024.1521634, PMID: 39845946 PMC11750673

[17] Liu X Wang Y Zhang Y Jiang H Huo X Liu R . Integrated bioinformatic analysis and experimental validation to reveal the mechanisms of xinfeng capsule against rheumatoid arthritis. Comb Chem High t scr. (2023) 26:2161–9. doi: 10.2174/1386207326666230127151049, PMID: 36705239

[18] Zhu Z Wan L . Exploration of the molecular mechanism guiding Xinfeng capsule regulatory mechanism for rheumatoid arthritis inflammation. Am J Transl Res. (2024) 16:973–87. doi: 10.62347/TPOQ4910, PMID: 38586085 PMC10994809

[19] Wang F Liu J Wang Y Sun Y Wen J He M . Correlation analysis of coagulation and platelet parameters with clinical outcomes in rheumatoid arthritis patients and the interventional effect of jianpi huashi tongluo formula - xinfeng capsule: A *post hoc* analysis based on an randomized controlled trial. Drug Des Devel Ther. (2025) 19:3477–95. doi: 10.2147/DDDT.S512338, PMID: 40322027 PMC12049676

[20] Wang F Liu J Fang Y Sun Y He M . The treatment with xinfeng capsule can reduce the risk of readmission for patients with rheumatoid arthritis:A cohort study of approximately 10000 individuals. Int J Gen Med. (2024) 17:5285–98. doi: 10.2147/IJGM.S491218, PMID: 39563785 PMC11575443

[21] Sun Y Liu J Xin L Wen J Zhou Q Chen X . Xinfeng capsule inhibits inflammation and oxidative stress in rheumatoid arthritis by up-regulating LINC00638 and activating Nrf2/HO-1 pathway. J Ethnopharmacol. (2023) 301:115839. doi: 10.1016/j.jep.2022.115839, PMID: 36272490

[22] Fu W Cao Y Liu J Huang C Shu K Zhu N . Xinfeng capsule inhibits pyroptosis and ameliorates myocardial injury in rats with adjuvant arthritis via the GAS5/miR-21/TLR4 axis. Drug Des Devel Ther. (2024) 18:2421–33. doi: 10.2147/DDDT.S456783, PMID: 38915862 PMC11195676

[23] Gao L Wang F Meng M . Chromatographic fingerprinting and quantitative analysis for the quality evaluation of Xinfeng capsule. Acta Chromatographica AChrom. (2020) 33:37–43. doi: 10.1556/1326.2020.00743, PMID: 29403777

[24] Aletaha D Neogi T Silman AJ Funovits J Felson DT Bingham CO . 2010 rheumatoid arthritis classification criteria: an American College of Rheumatology/European League Against Rheumatism collaborative initiative. Ann Rheum Dis. (2010) 69:1580–8. doi: 10.1136/ard.2010.138461, PMID: 20699241

[25] Langworthy B Wu Y Wang M . An overview of propensity score matching methods for clustered data. Stat Methods Med Res. (2023) 32:641–55. doi: 10.1177/09622802221133556, PMID: 36426585 PMC10119899

[26] Fang Y Liu J Xin L Sun Y Wan L Huang D . Identifying compound effect of drugs on rheumatoid arthritis treatment based on the association rule and a random walking-based model. BioMed Res Int. (2020) 2020:4031015. doi: 10.1155/2020/4031015, PMID: 33204694 PMC7665920

[27] Demkow U . Molecular mechanisms of neutrophil extracellular trap (NETs) degradation. Int J Mol Sci. (2023) 24:4896. doi: 10.3390/ijms24054896, PMID: 36902325 PMC10002918

[28] Lee HT Lin CS Liu CY Chen P Tsai CY Wei YH . Mitochondrial plasticity and glucose metabolic alterations in human cancer under oxidative stress-from viewpoints of chronic inflammation and neutrophil extracellular traps (NETs). Int J Mol Sci. (2024) 25:9485. doi: 10.3390/ijms25179458, PMID: 39273403 PMC11395599

[29] Li Y Liu J Sun Y Hu Y Cong C Chen Y . Targeting p38 MAPK signaling pathway and neutrophil extracellular traps: An important anti-inflammatory mechanism of Huangqin Qingre Chubi Capsule in rheumatoid arthritis. Int Immunopharmacol. (2025) 148:114112. doi: 10.1016/j.intimp.2025.114112, PMID: 39837014

[30] Wen J Liu J Zhang P Jiang H Xin L Wan L . RNA-seq reveals the circular RNA and miRNA expression profile of peripheral blood mononuclear cells in patients with rheumatoid arthritis. Bioscience Rep. (2020) 40:BSR20193160. doi: 10.1042/BSR20193160, PMID: 32191279 PMC7133114

[31] Luo Q Gao Y Zhang L Rao J Guo Y Huang Z . Decreased ALKBH5, FTO, and YTHDF2 in peripheral blood are as risk factors for rheumatoid arthritis. BioMed Res Int. (2020) 2020:5735279. doi: 10.1155/2020/5735279, PMID: 32884942 PMC7455827

[32] Payet M Dargai F Gasque P Guillot X . Epigenetic regulation (Including micro-RNAs, DNA methylation and histone modifications) of rheumatoid arthritis: A systematic review. Int J Mol Sci. (2021) 22:12170. doi: 10.3390/ijms222212170, PMID: 34830057 PMC8625518

[33] Li Y Liu J Hu Y Cong C Chen Y Fang Y . The neutrophil-to-lymphocyte ratio in rheumatoid arthritis: The dual perspectives from literature and clinic. Med (Baltimore). (2025) 104:e44554. doi: 10.1097/MD.0000000000044554, PMID: 40988164 PMC12459527

[34] Song BW Kim AR Kim YK Kim GT Ahn EY So MW . Diagnostic value of neutrophil-to-lymphocyte, platelet-to-lymphocyte, and monocyte-to-lymphocyte ratios for the assessment of rheumatoid arthritis in patients with undifferentiated inflammatory arthritis. Diagnostics (Basel). (2022) 12:1702. doi: 10.3390/diagnostics12071702, PMID: 35885606 PMC9317908

[35] Liu B Wang J Li YY Li KP Zhang Q . The association between systemic immune-inflammation index and rheumatoid arthritis: evidence from NHANES 1999-2018. Arthritis Res Ther. (2023) 25:34. doi: 10.1186/s13075-023-03018-6, PMID: 36871051 PMC9985219

[36] Sun Y Liu J Wang Y Wan L Huang D Li Y . Efficacy analysis of xinfeng capsule in modulating immune inflammatory levels in patients with rheumatoid arthritis presenting with damp-heat patterns. Chin J Clin. (2024) 52:1126–30. doi: 10.3969/j.issn.2095-8552.2024.09.033

[37] Yin F Hong H Wang Y Wang Y Zhang J Tang X . Mechanistic insights into NETs-induced osteogenesis inhibition in BMSCs of rheumatoid arthritis. Bone. (2025) 198:117533. doi: 10.1016/j.bone.2025.117533, PMID: 40414474

[38] Zhang J Xie X Shen Q Yuan C Lu G Xiao W . Rhaponticin alleviates collagen-induced arthritis by inhibiting NLRP3/GSDMD-mediated neutrophil extracellular t’raps. Inflammation. (2025) 48:2756–71. doi: 10.1007/s10753-024-02228-7, PMID: 39725843 PMC12336096

[39] Wysocki T Wajda A Kmiołek T Wroński J Roszkowska M Olesińska M . NADPH oxidase expression profile and PBMC immunophenotypic changes in anti-TNF-treated rheumatoid arthritis patients. Clin Immunol. (2025) 271:110414. doi: 10.1016/j.clim.2024.110414, PMID: 39643026

[40] Smallwood MJ Nissim A Knight AR Whiteman M Haigh R Winyard PG . Oxidative stress in autoimmune rheumatic diseases. Free Radic Biol Med. (2018) 125:3–14. doi: 10.1016/j.freeradbiomed.2018.05.086, PMID: 29859343

[41] Larosa V Remacle C . Insights into the respiratory chain and oxidative stress. Bioscience Rep. (2018) 38:BSR20171492. doi: 10.1042/BSR20171492, PMID: 30201689 PMC6167499

[42] Yang Q Liu R Yu Q Bi Y Liu G . Metabolic regulation of inflammasomes in inflammation. Immunology. (2019) 157:95–109. doi: 10.1111/imm.13056, PMID: 30851192 PMC6526672

[43] Wang Z Dong Q Zhang L Wang X Jiao Y Geng Q . Fibroblast activation protein-α drives rheumatoid arthritis inflammation through the AKT/mTOR signaling pathway and its therapeutic effect by Wangbi granules. Phytomedicine. (2025) 147:157179. doi: 10.1016/j.phymed.2025.157179, PMID: 40857963

[44] Wang F Wen J Liu J Xin L Fang Y Sun Y . Demethylase FTO mediates m6A modification of ENST00000619282 to promote apoptosis escape in rheumatoid arthritis and the intervention effect of Xinfeng Capsule. Front Immunol. (2025) 16:1556764. doi: 10.3389/fimmu.2025.1556764, PMID: 40181982 PMC11966437

