# Supplementary figures and images for: Unveiling novel therapeutic mechanisms of Xinfeng capsule: modulating the ALKBH5–m6A–LINC00968 axis to alleviate oxidative stress-driven NETosis in rheumatoid arthritis
Source: Front Immunol. 2025 Dec 17;16:1707663. doi: 10.3389/fimmu.2025.1707663 (PMC12753411; doi:10.3389/fimmu.2025.1707663)

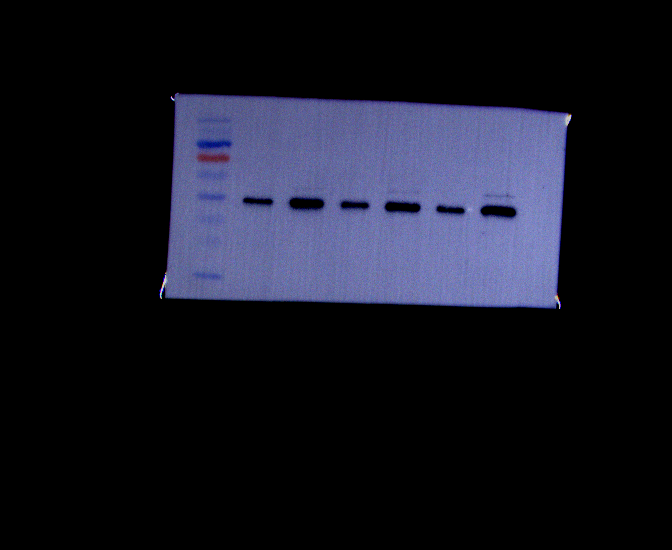

Supplement: Supplementary file 1 [file DataSheet1.zip › ID 1707663_WB original images/Figure 2F/Figure 2F_ALKBH5.tif]

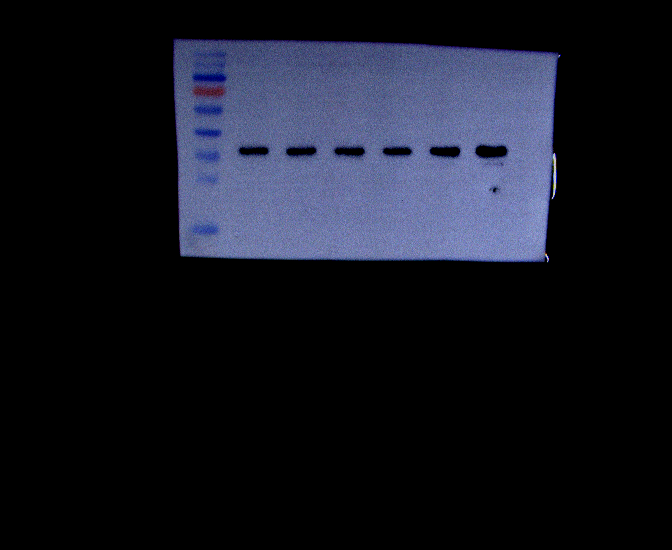

Supplement: Supplementary file 1 [file DataSheet1.zip › ID 1707663_WB original images/Figure 2F/Figure 2F_GAPDH.tif]

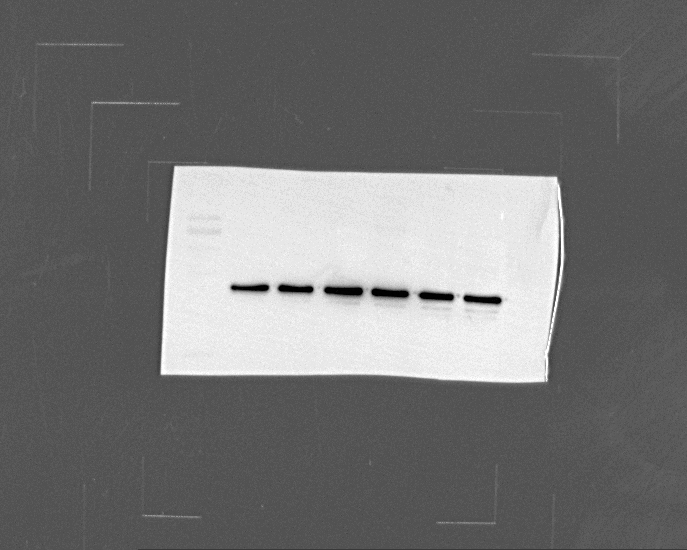

Supplement: Supplementary file 1 [file DataSheet1.zip › ID 1707663_WB original images/Figure 4I/Figure 4I_GAPDH_1.tif]

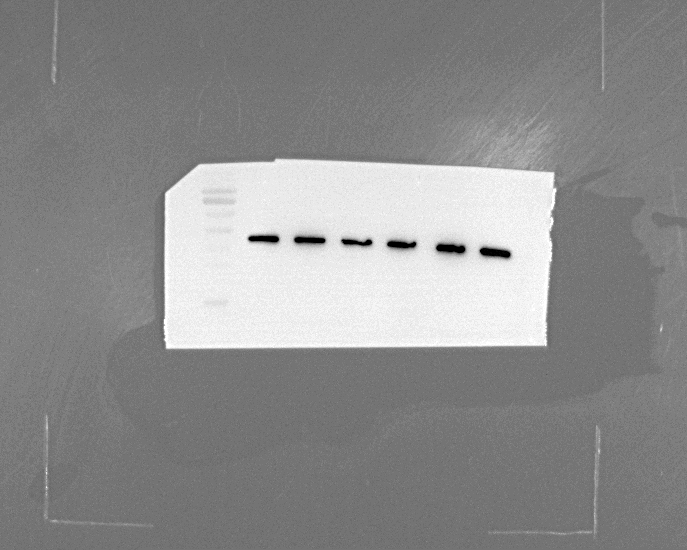

Supplement: Supplementary file 1 [file DataSheet1.zip › ID 1707663_WB original images/Figure 4I/Figure 4I_GAPDH_2.tif]

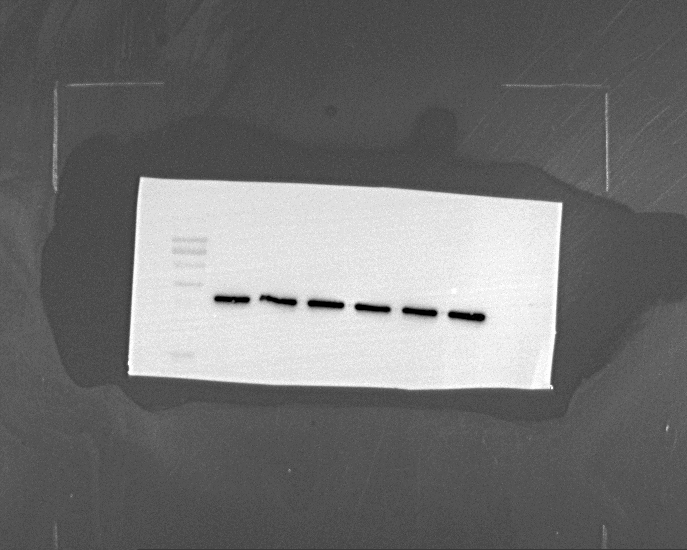

Supplement: Supplementary file 1 [file DataSheet1.zip › ID 1707663_WB original images/Figure 4I/Figure 4I_GAPDH_3.tif]

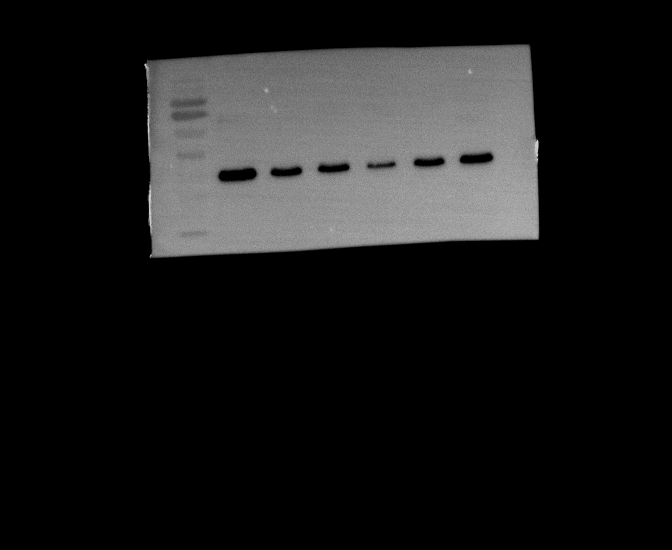

Supplement: Supplementary file 1 [file DataSheet1.zip › ID 1707663_WB original images/Figure 4I/Figure 4I_HO-1_1.tif]

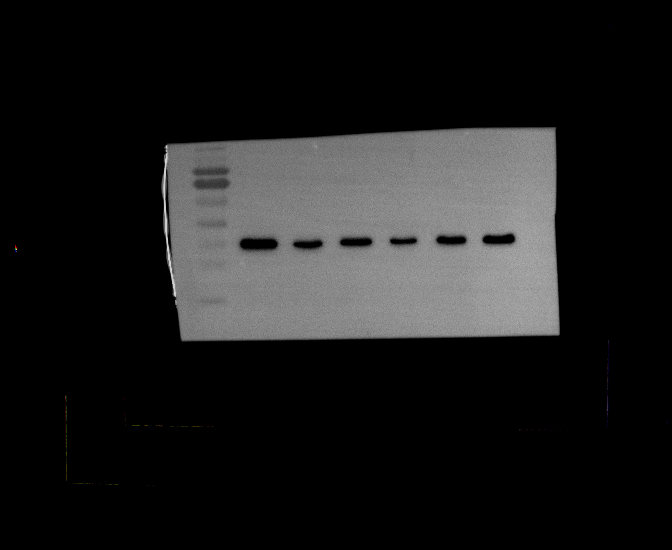

Supplement: Supplementary file 1 [file DataSheet1.zip › ID 1707663_WB original images/Figure 4I/Figure 4I_HO-1_2.tif]

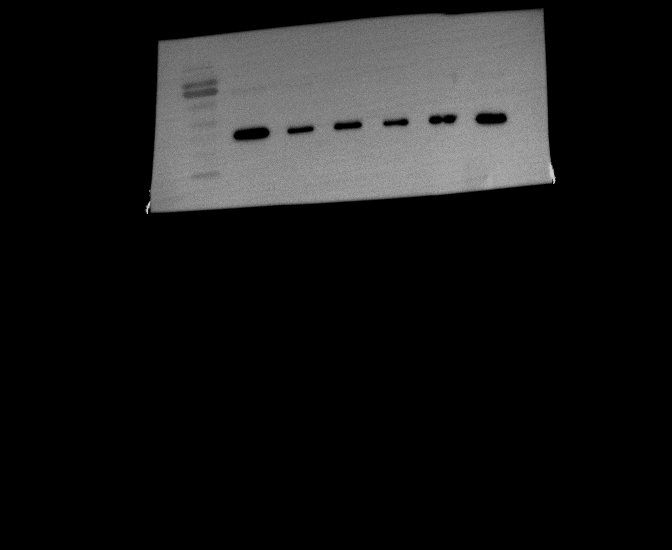

Supplement: Supplementary file 1 [file DataSheet1.zip › ID 1707663_WB original images/Figure 4I/Figure 4I_HO-1_3.tif]

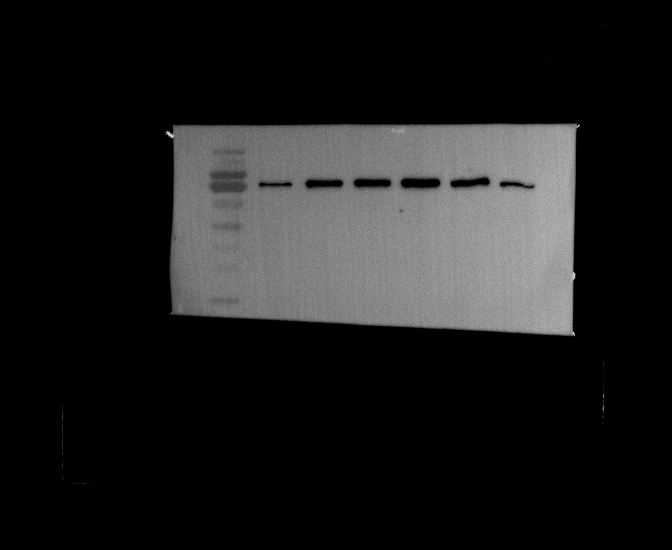

Supplement: Supplementary file 1 [file DataSheet1.zip › ID 1707663_WB original images/Figure 4I/Figure 4I_NOX_1.tif]

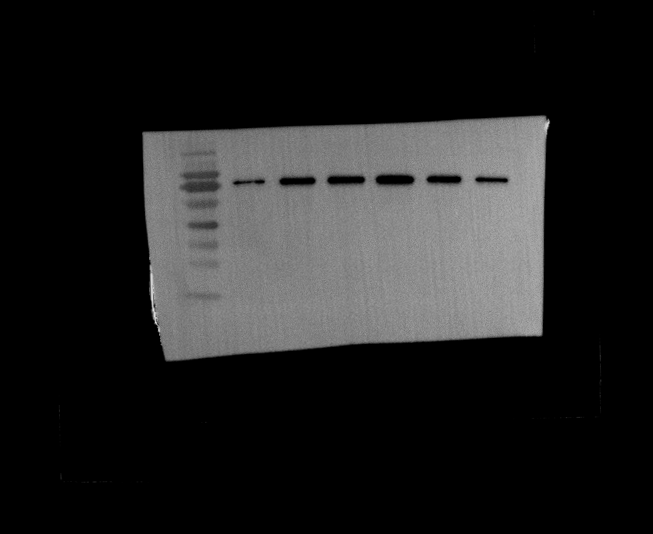

Supplement: Supplementary file 1 [file DataSheet1.zip › ID 1707663_WB original images/Figure 4I/Figure 4I_NOX_2.tif]

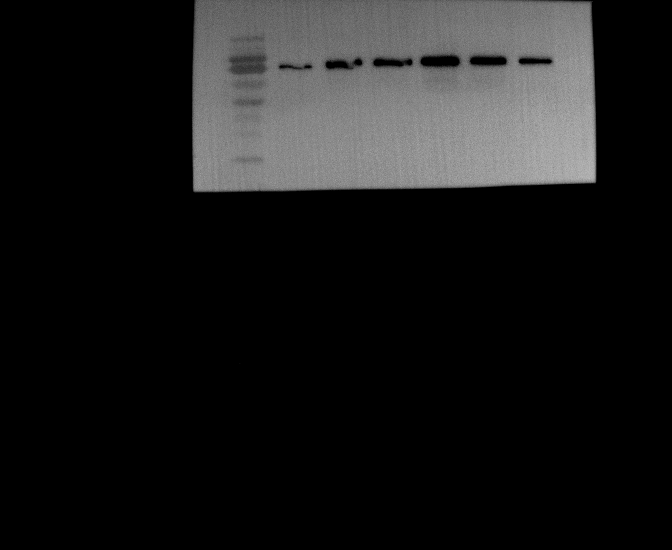

Supplement: Supplementary file 1 [file DataSheet1.zip › ID 1707663_WB original images/Figure 4I/Figure 4I_NOX_3.tif]

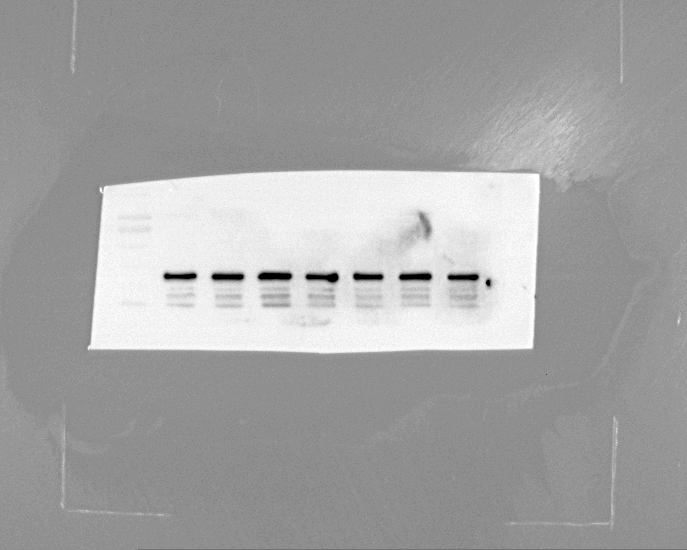

Supplement: Supplementary file 1 [file DataSheet1.zip › ID 1707663_WB original images/Figure 6I/Figure 6I_GAPDH_1.tif]

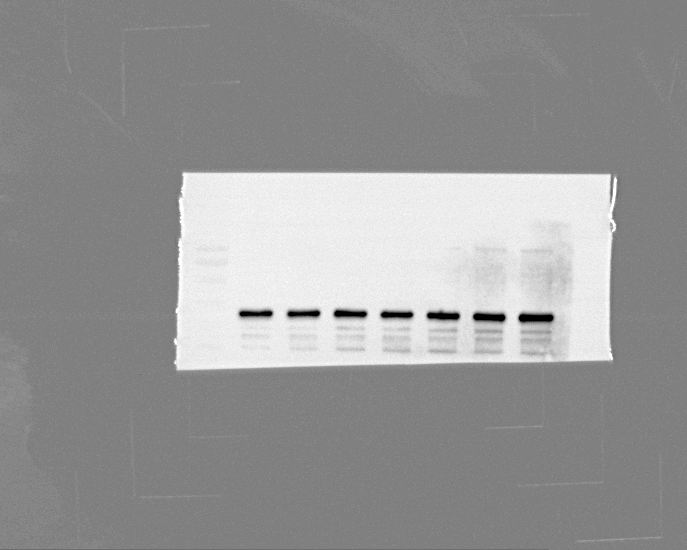

Supplement: Supplementary file 1 [file DataSheet1.zip › ID 1707663_WB original images/Figure 6I/Figure 6I_GAPDH_2.tif]

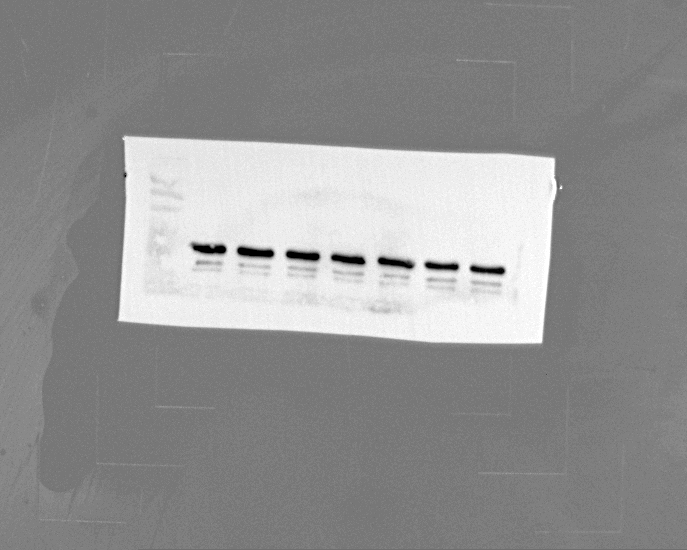

Supplement: Supplementary file 1 [file DataSheet1.zip › ID 1707663_WB original images/Figure 6I/Figure 6I_GAPDH_3.tif]

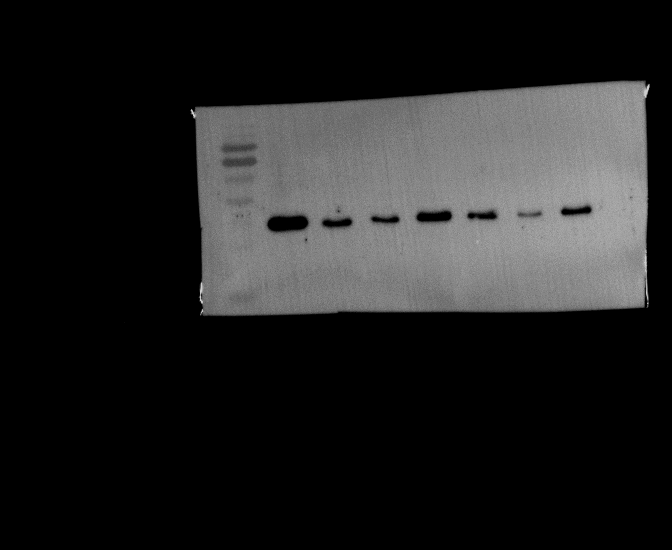

Supplement: Supplementary file 1 [file DataSheet1.zip › ID 1707663_WB original images/Figure 6I/Figure 6I_HO-1_1.tif]

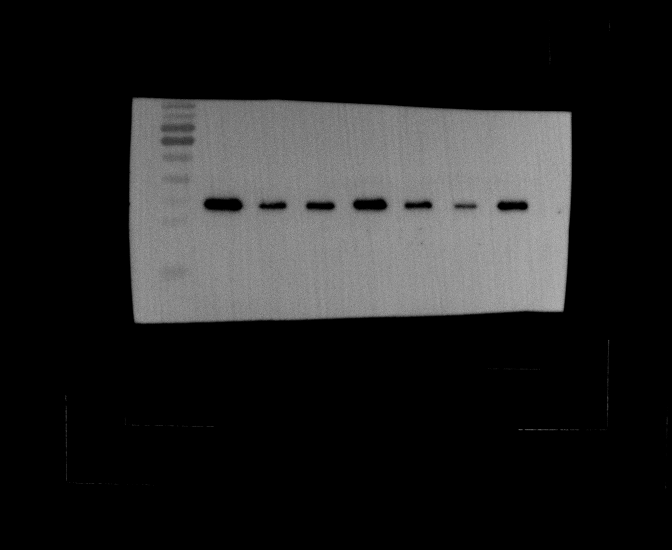

Supplement: Supplementary file 1 [file DataSheet1.zip › ID 1707663_WB original images/Figure 6I/Figure 6I_HO-1_2.tif]

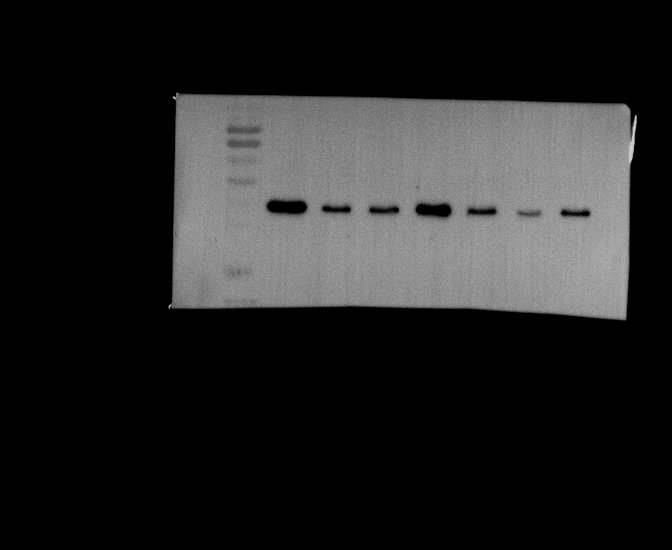

Supplement: Supplementary file 1 [file DataSheet1.zip › ID 1707663_WB original images/Figure 6I/Figure 6I_HO-1_3.tif]

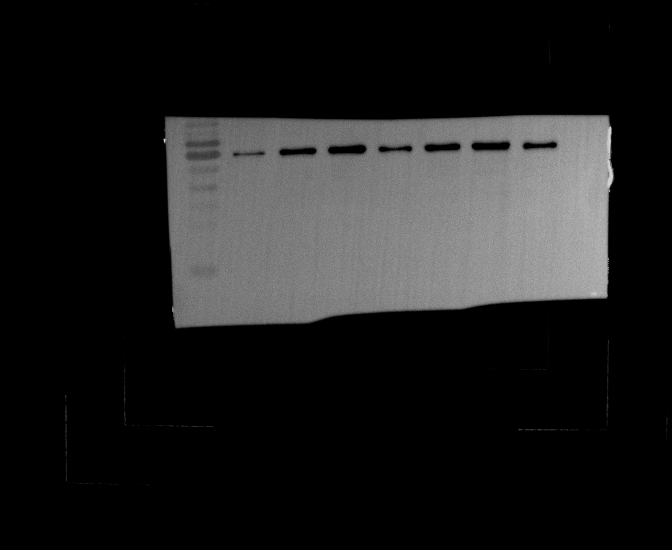

Supplement: Supplementary file 1 [file DataSheet1.zip › ID 1707663_WB original images/Figure 6I/Figure 6I_NOX_1.tif]

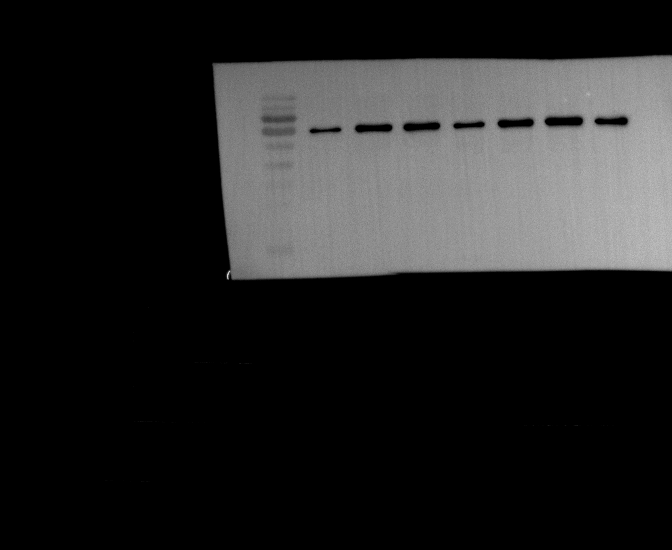

Supplement: Supplementary file 1 [file DataSheet1.zip › ID 1707663_WB original images/Figure 6I/Figure 6I_NOX_2.tif]

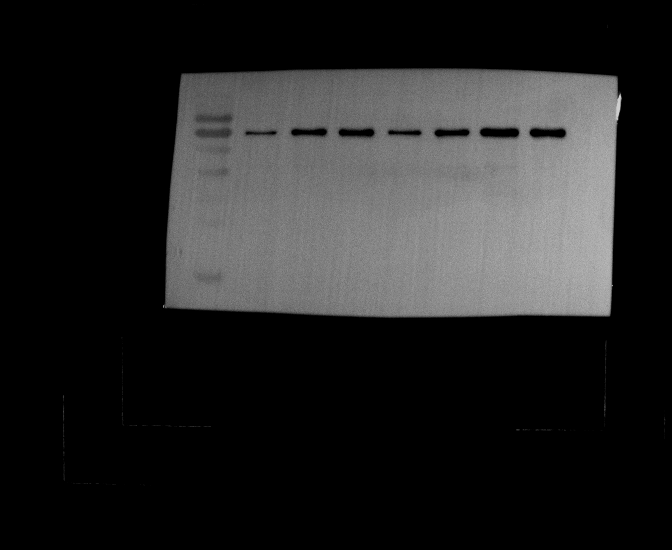

Supplement: Supplementary file 1 [file DataSheet1.zip › ID 1707663_WB original images/Figure 6I/Figure 6I_NOX_3.tif]

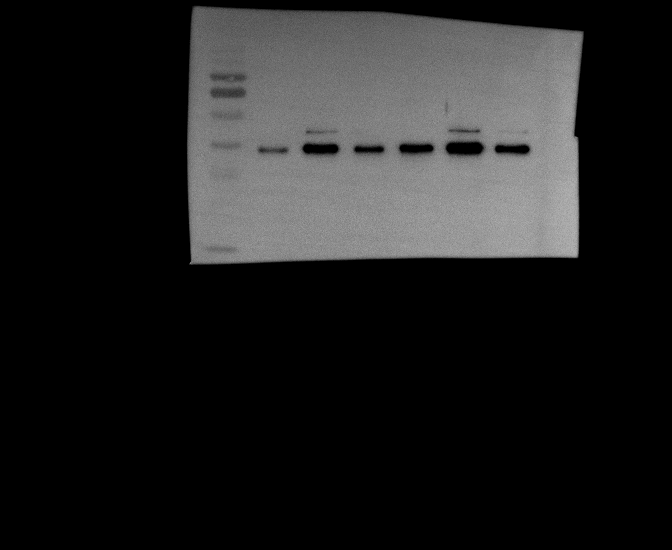

Supplement: Supplementary file 1 [file DataSheet1.zip › ID 1707663_WB original images/Figure 8I/Figure 8I_ALKBH5_1.tif]

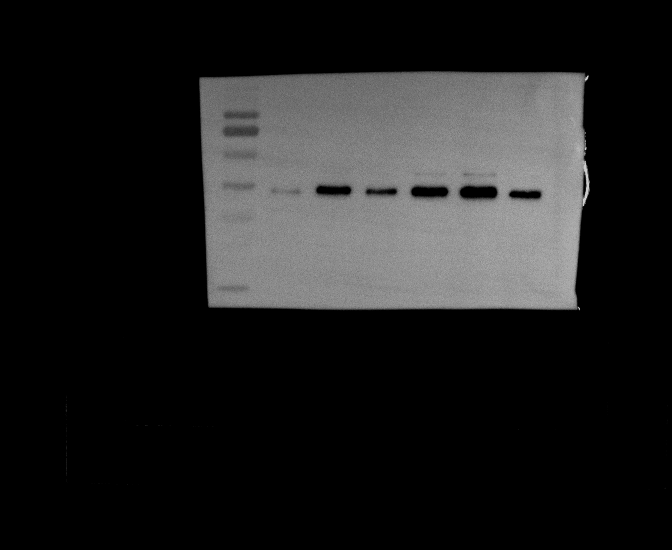

Supplement: Supplementary file 1 [file DataSheet1.zip › ID 1707663_WB original images/Figure 8I/Figure 8I_ALKBH5_2.tif]

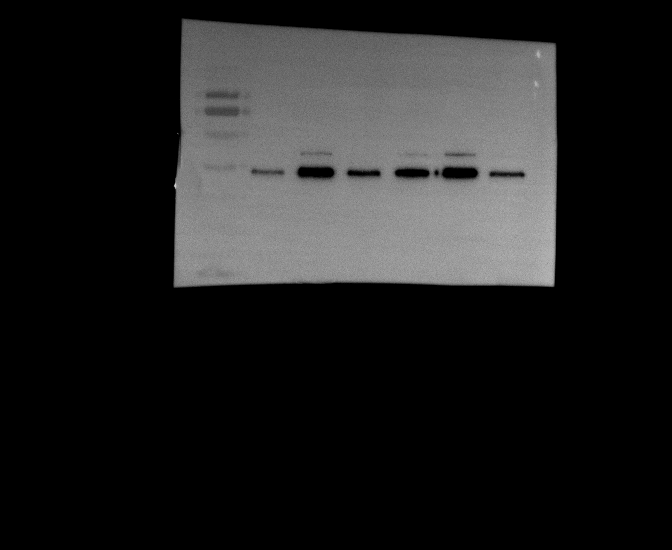

Supplement: Supplementary file 1 [file DataSheet1.zip › ID 1707663_WB original images/Figure 8I/Figure 8I_ALKBH5_3.tif]

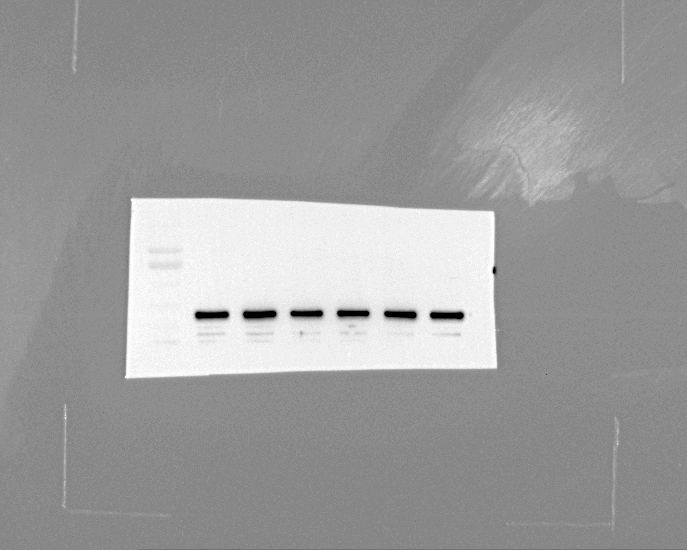

Supplement: Supplementary file 1 [file DataSheet1.zip › ID 1707663_WB original images/Figure 8I/Figure 8I_GAPDH_1.tif]

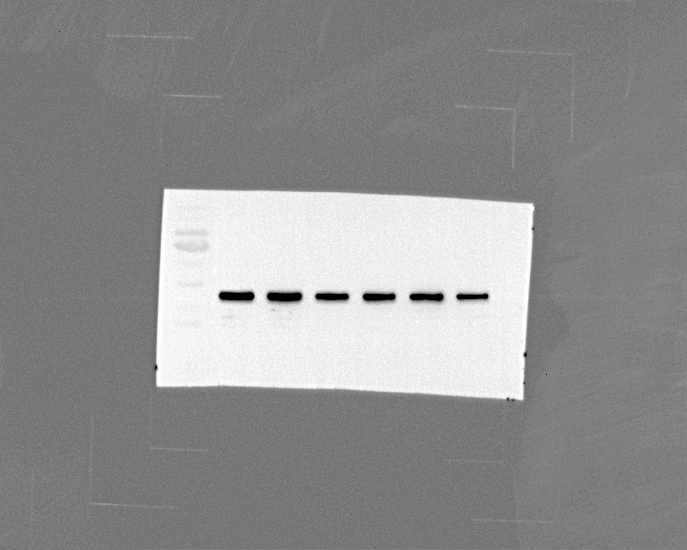

Supplement: Supplementary file 1 [file DataSheet1.zip › ID 1707663_WB original images/Figure 8I/Figure 8I_GAPDH_2.tif]

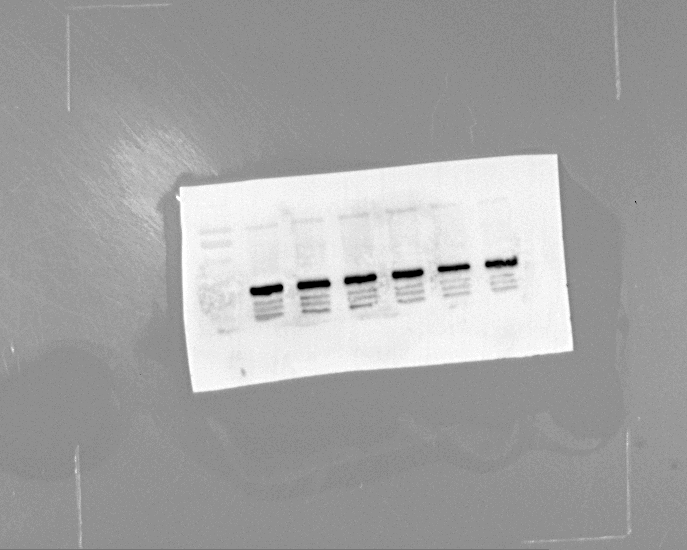

Supplement: Supplementary file 1 [file DataSheet1.zip › ID 1707663_WB original images/Figure 8I/Figure 8I_GAPDH_3.tif]

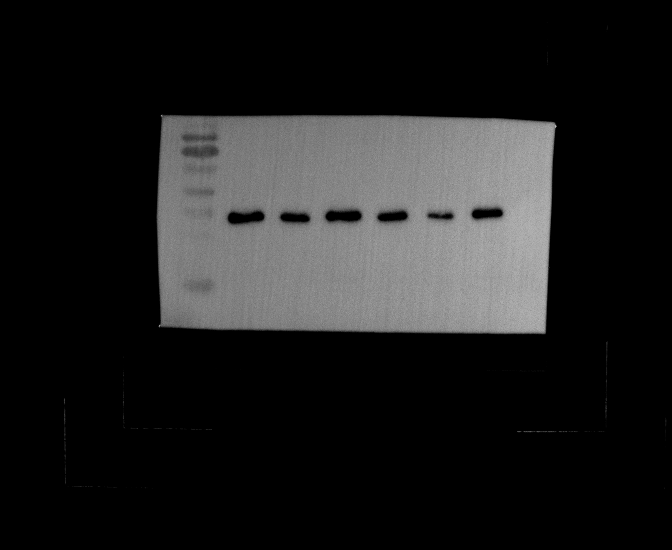

Supplement: Supplementary file 1 [file DataSheet1.zip › ID 1707663_WB original images/Figure 8I/Figure 8I_HO-1_1.tif]

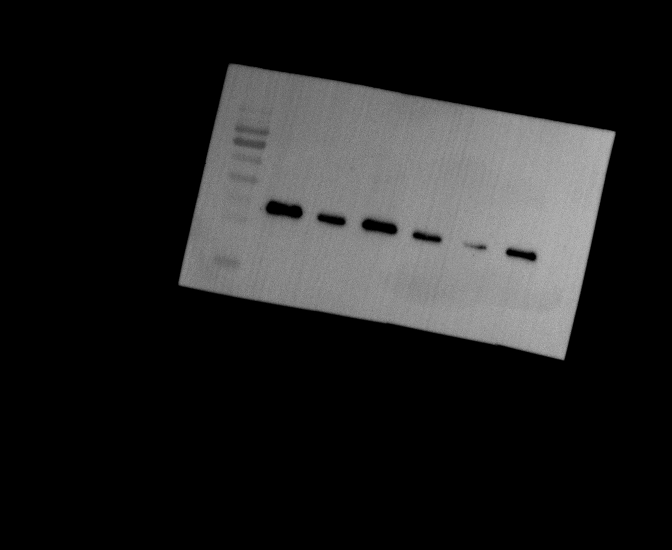

Supplement: Supplementary file 1 [file DataSheet1.zip › ID 1707663_WB original images/Figure 8I/Figure 8I_HO-1_2.tif]

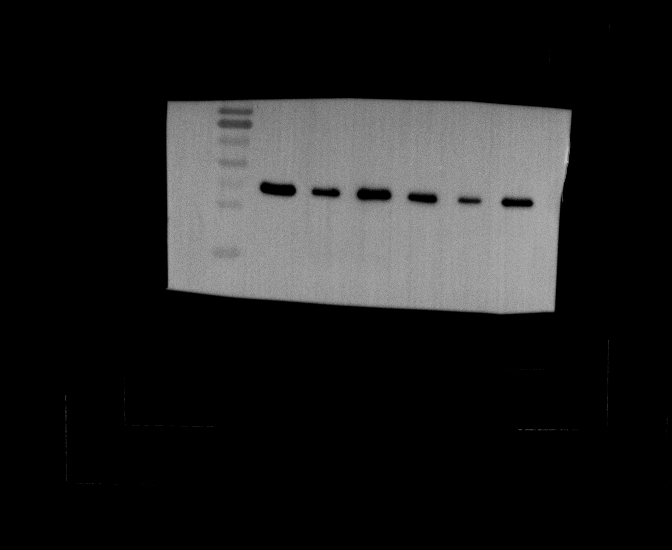

Supplement: Supplementary file 1 [file DataSheet1.zip › ID 1707663_WB original images/Figure 8I/Figure 8I_HO-1_3.tif]

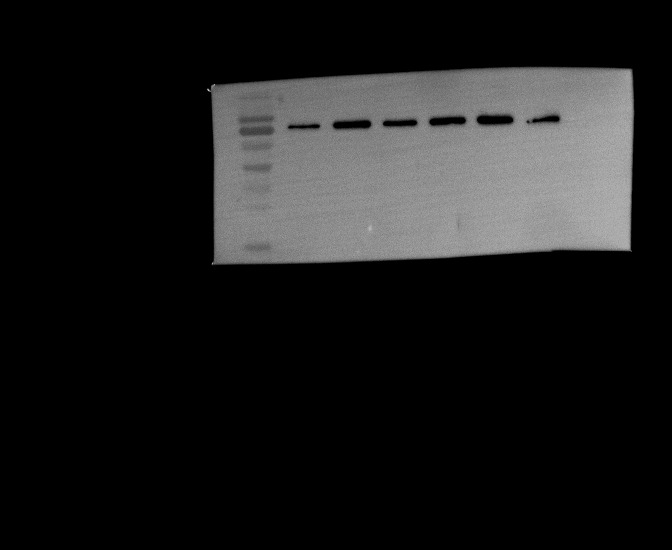

Supplement: Supplementary file 1 [file DataSheet1.zip › ID 1707663_WB original images/Figure 8I/Figure 8I_NOX_1.tif]

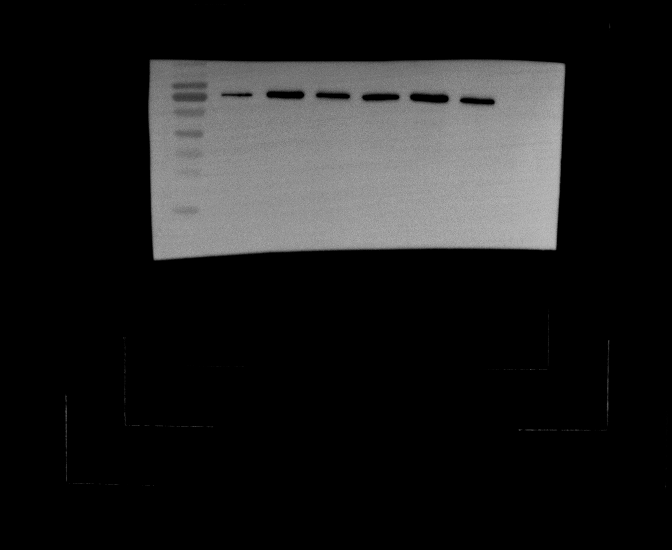

Supplement: Supplementary file 1 [file DataSheet1.zip › ID 1707663_WB original images/Figure 8I/Figure 8I_NOX_2.tif]

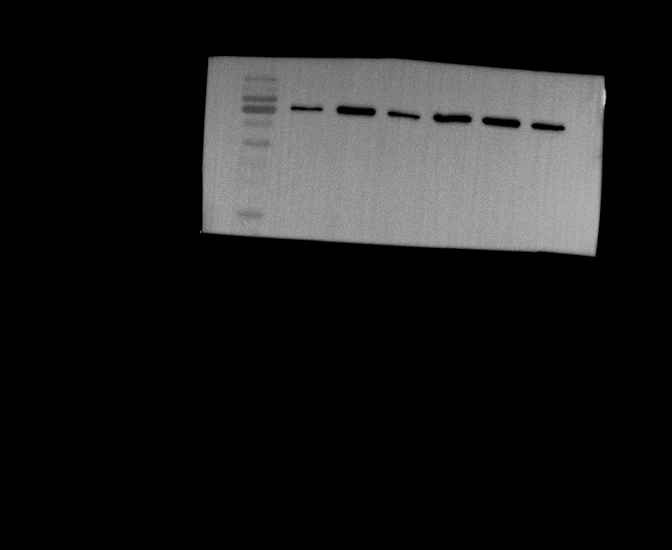

Supplement: Supplementary file 1 [file DataSheet1.zip › ID 1707663_WB original images/Figure 8I/Figure 8I_NOX_3.tif]

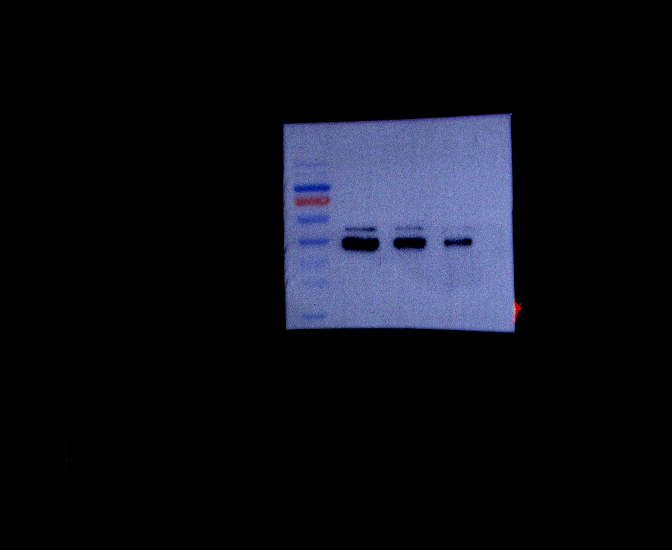

Supplement: Supplementary file 1 [file DataSheet1.zip › ID 1707663_WB original images/Figure 10I/Figure 10I_ALKBH5_1.tif]

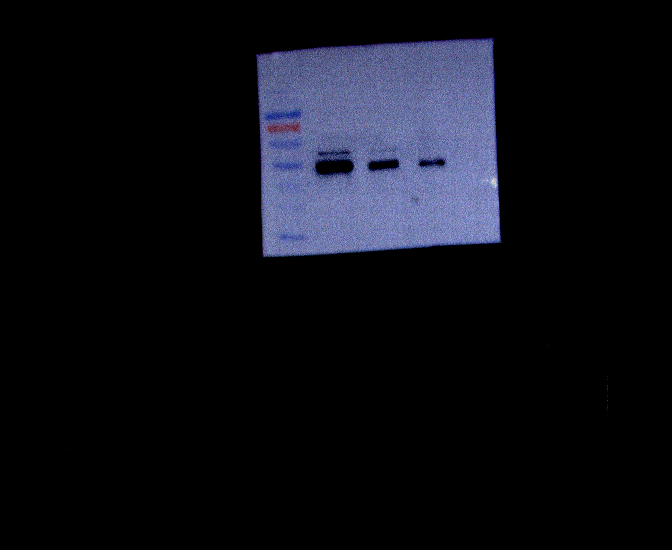

Supplement: Supplementary file 1 [file DataSheet1.zip › ID 1707663_WB original images/Figure 10I/Figure 10I_ALKBH5_2.tif]

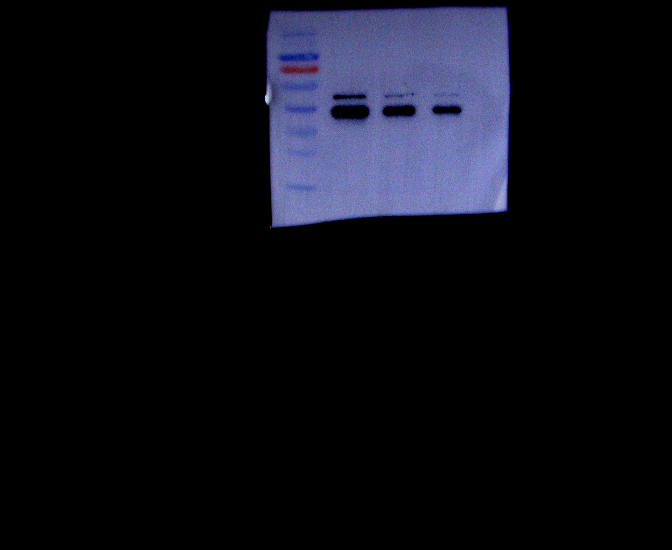

Supplement: Supplementary file 1 [file DataSheet1.zip › ID 1707663_WB original images/Figure 10I/Figure 10I_ALKBH5_3.tif]

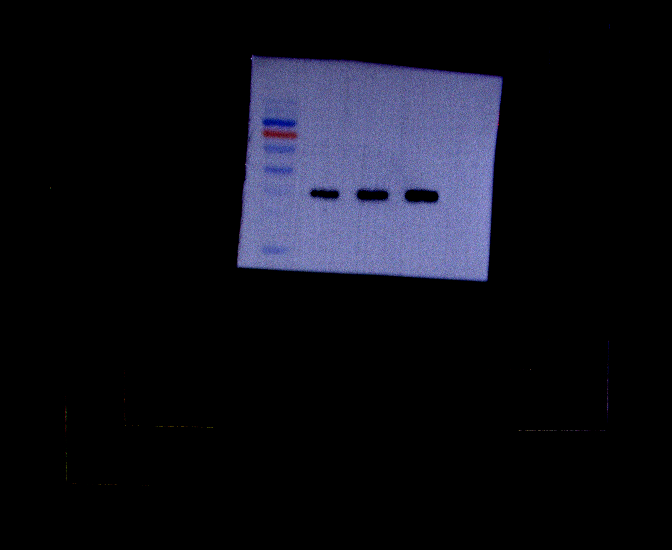

Supplement: Supplementary file 1 [file DataSheet1.zip › ID 1707663_WB original images/Figure 10I/Figure 10I_HO-1_1.tif]

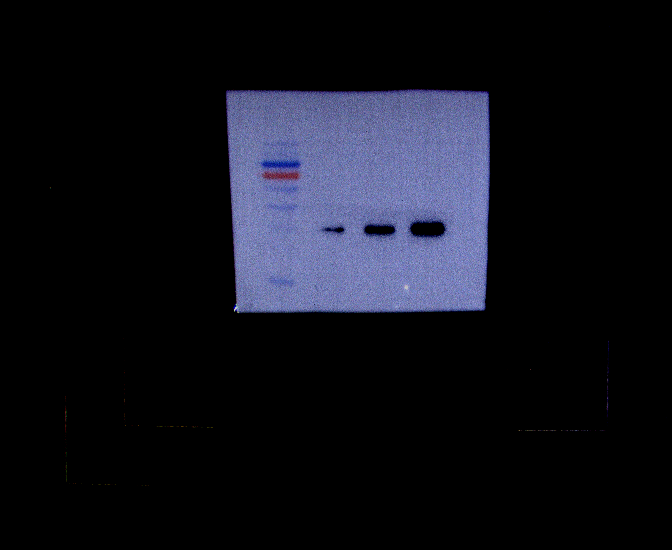

Supplement: Supplementary file 1 [file DataSheet1.zip › ID 1707663_WB original images/Figure 10I/Figure 10I_HO-1_2.tif]

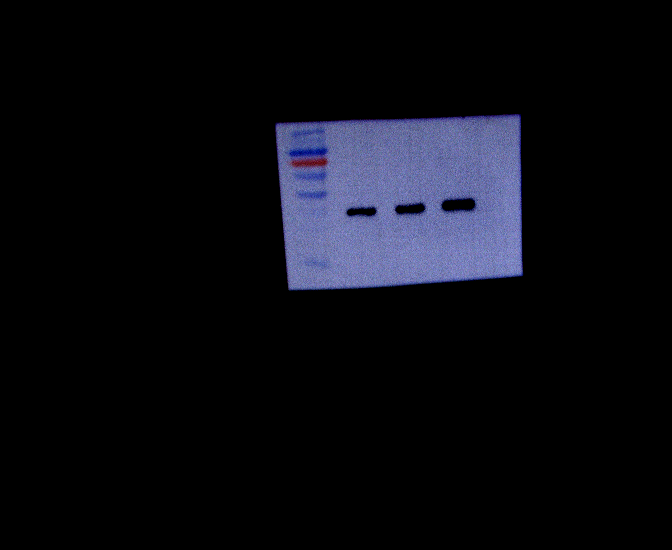

Supplement: Supplementary file 1 [file DataSheet1.zip › ID 1707663_WB original images/Figure 10I/Figure 10I_HO-1_3.tif]

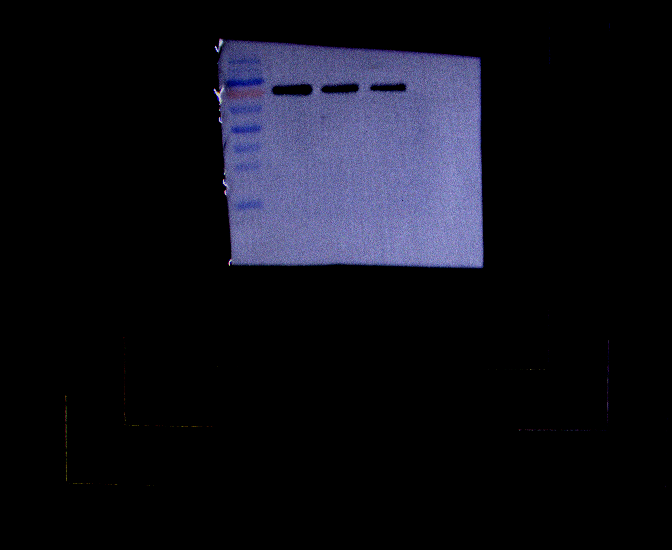

Supplement: Supplementary file 1 [file DataSheet1.zip › ID 1707663_WB original images/Figure 10I/Figure 10I_NOX_1.tif]

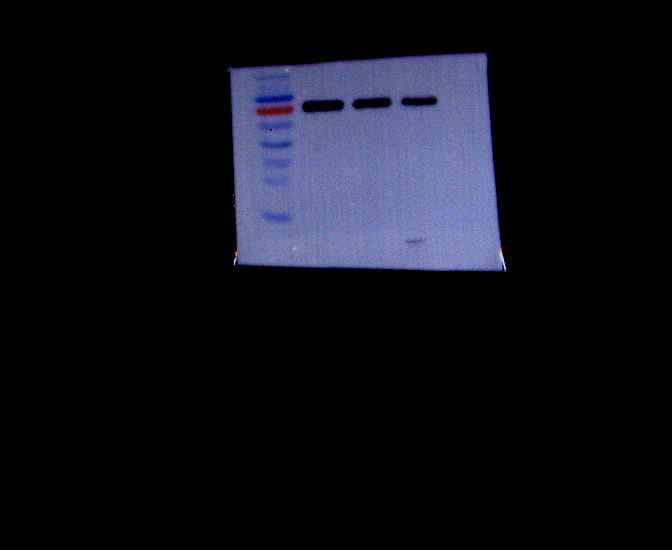

Supplement: Supplementary file 1 [file DataSheet1.zip › ID 1707663_WB original images/Figure 10I/Figure 10I_NOX_2.tif]

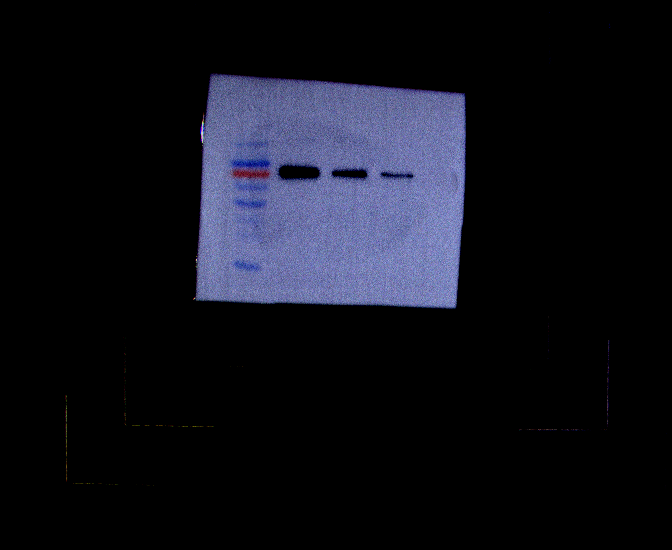

Supplement: Supplementary file 1 [file DataSheet1.zip › ID 1707663_WB original images/Figure 10I/Figure 10I_NOX_3.tif]

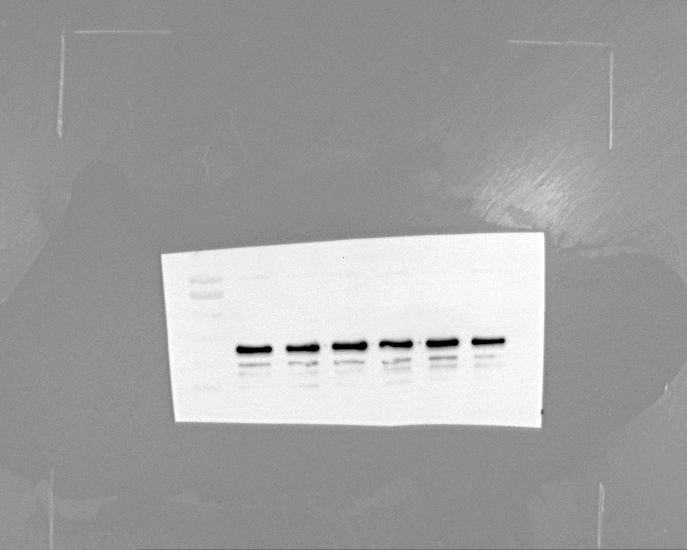

Supplement: Supplementary file 1 [file DataSheet1.zip › ID 1707663_WB original images/Figure 12I/Figure 12I_GAPDH_1.tif]

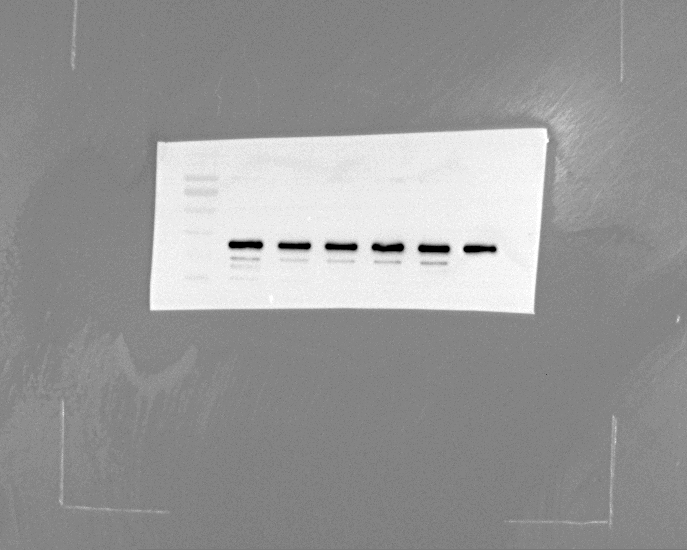

Supplement: Supplementary file 1 [file DataSheet1.zip › ID 1707663_WB original images/Figure 12I/Figure 12I_GAPDH_2.tif]

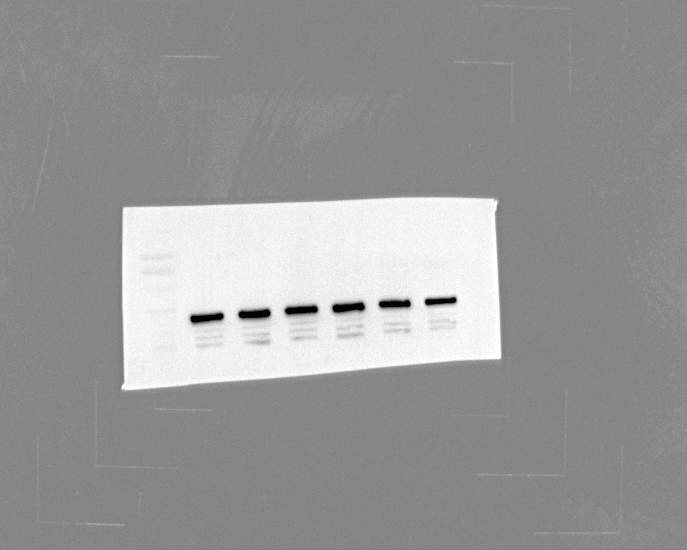

Supplement: Supplementary file 1 [file DataSheet1.zip › ID 1707663_WB original images/Figure 12I/Figure 12I_GAPDH_3.tif]

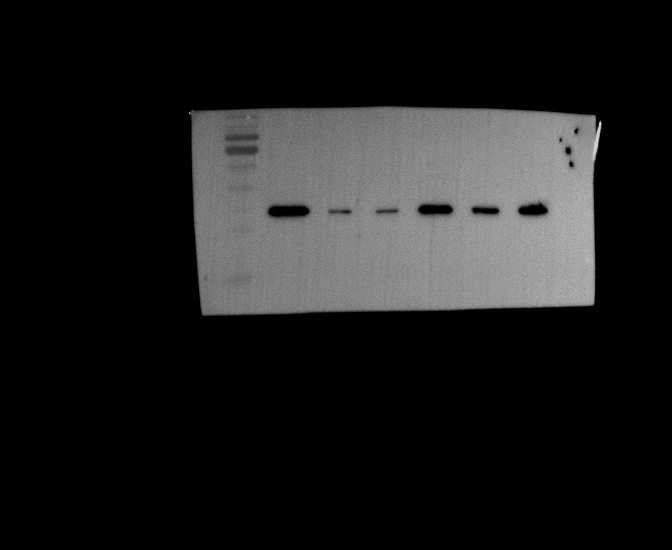

Supplement: Supplementary file 1 [file DataSheet1.zip › ID 1707663_WB original images/Figure 12I/Figure 12I_HO-1_1.tif]

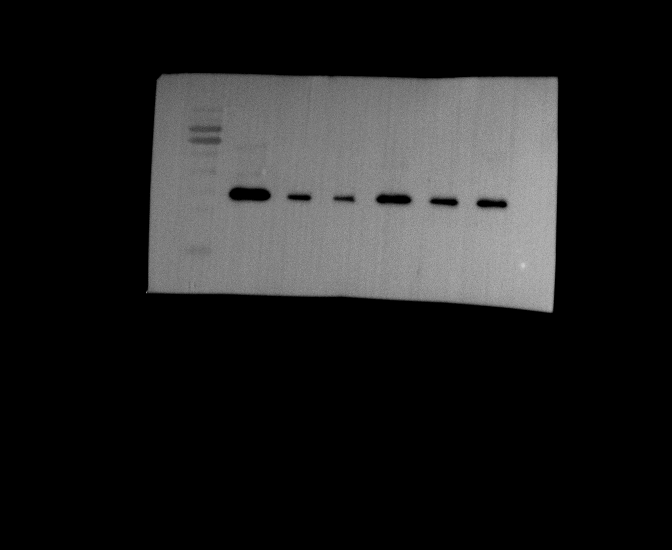

Supplement: Supplementary file 1 [file DataSheet1.zip › ID 1707663_WB original images/Figure 12I/Figure 12I_HO-1_2.tif]

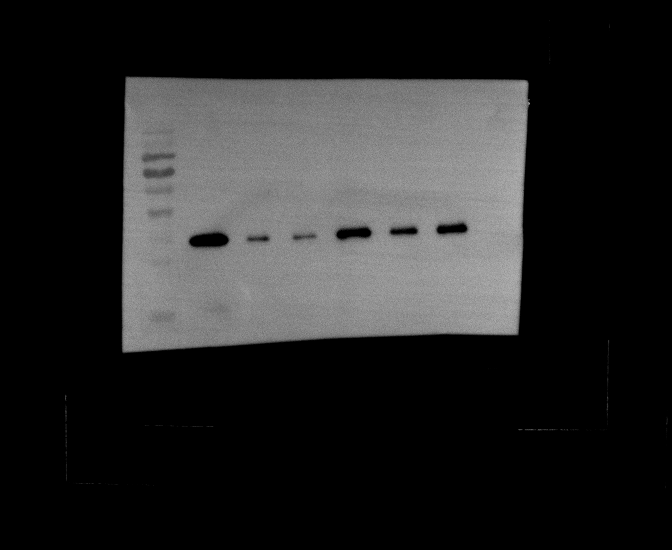

Supplement: Supplementary file 1 [file DataSheet1.zip › ID 1707663_WB original images/Figure 12I/Figure 12I_HO-1_3.tif]

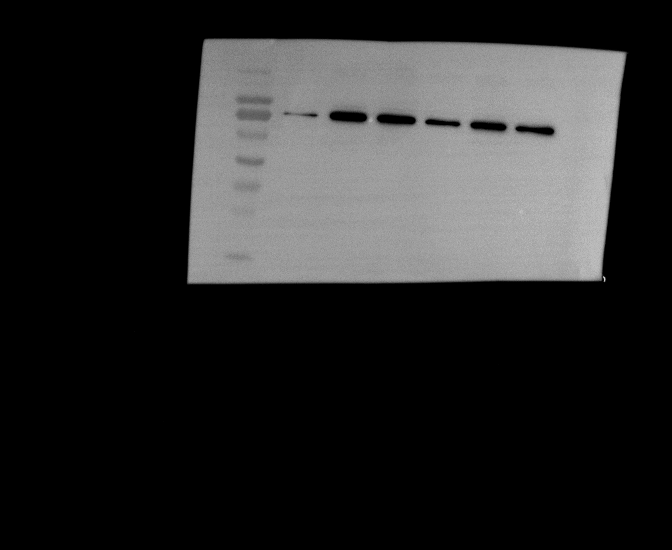

Supplement: Supplementary file 1 [file DataSheet1.zip › ID 1707663_WB original images/Figure 12I/Figure 12I_NOX_1.tif]

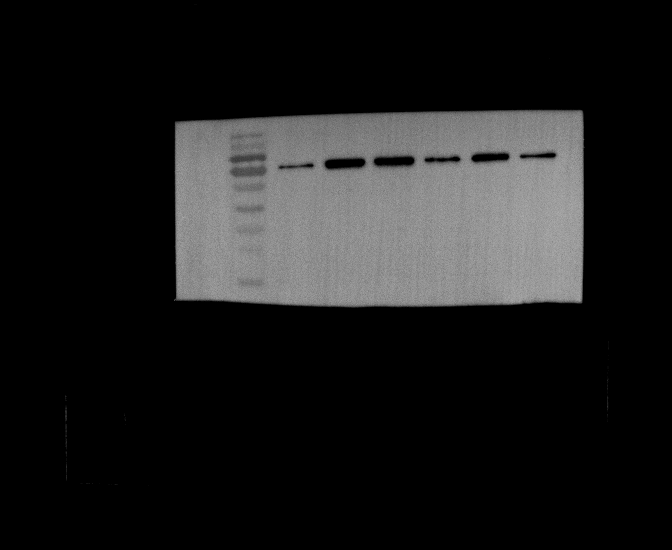

Supplement: Supplementary file 1 [file DataSheet1.zip › ID 1707663_WB original images/Figure 12I/Figure 12I_NOX_2.tif]

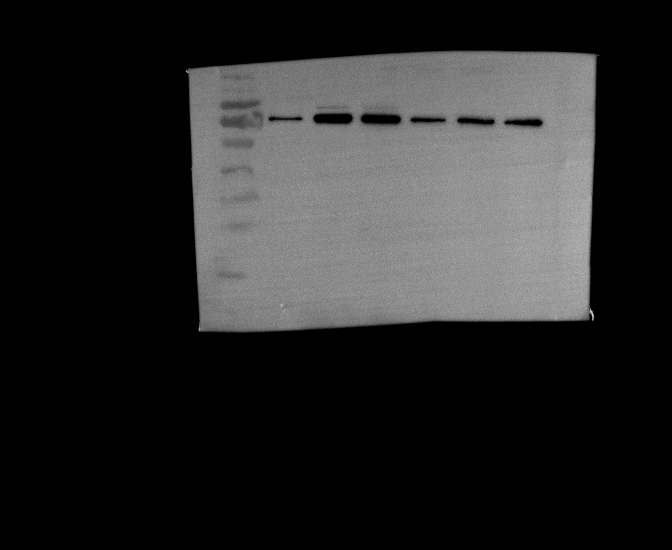

Supplement: Supplementary file 1 [file DataSheet1.zip › ID 1707663_WB original images/Figure 12I/Figure 12I_NOX_3.tif]
