# Supplementary material for: Unveiling novel therapeutic mechanisms of Xinfeng capsule: modulating the ALKBH5–m6A–LINC00968 axis to alleviate oxidative stress-driven NETosis in rheumatoid arthritis
Source: Front Immunol. 2025 Dec 17;16:1707663. doi: 10.3389/fimmu.2025.1707663 (PMC12753411; doi:10.3389/fimmu.2025.1707663)
Supplement: Supplementary file 2 [file DataSheet2.pdf]

frontiers

The International Society for the Advancement of Cytometry (ISAC) has highlighted the importance of including comprehensive methodological information to ensure data reproducibility and reliability. In line with this, Frontiers in Immunology now requires authors to submit a checklist for manuscripts that involve flow or mass cytometry. This checklist helps standardize the reporting process, improving the quality and transparency of published data. By doing so, we support scientific progress, making it easier for other researchers to replicate and validate experiments.

This form should be submitted with any manuscripts using flow or mass cytometry.

Sample/specimen/material description

☐ Total blood

☐ PBMCs

☐ Organ digests

Other Neutrophils

Did the samples suffer any treatment before or after incubation with the antibodies?

☐ Drug ______

☐ Cell permeabilization ______

☐ Dye ______

☐ Propidium iodine

☐ Not applicable

Other Ethanol

Instrument and antibodies

Name of the Cytometer Flow Cytometer(CytoFLEX, Beckman)

**Antibodies and targets**	**Fluorochrome/ Metal**	**Catalog number/Company**
e.g. anti-CD4	FITC	Cat. XXX/ XXX ltd.
BD Pharmingen™ PI/RNase Staining Buffer	PE	550825/BD Biosciences

frontiers

Data analyses

1. Name of the software Kaluza Analysis__________
2. Reference gating strategy in the manuscript or supplementary material

Gating strategy in (eg Figure X) Supplementary Figure 1______
