# Supplementary material for: Unveiling novel therapeutic mechanisms of Xinfeng capsule: modulating the ALKBH5–m6A–LINC00968 axis to alleviate oxidative stress-driven NETosis in rheumatoid arthritis
Source: Front Immunol. 2025 Dec 17;16:1707663. doi: 10.3389/fimmu.2025.1707663 (PMC12753411; doi:10.3389/fimmu.2025.1707663)
Supplement: Supplementary file 3 [file Table1.docx]

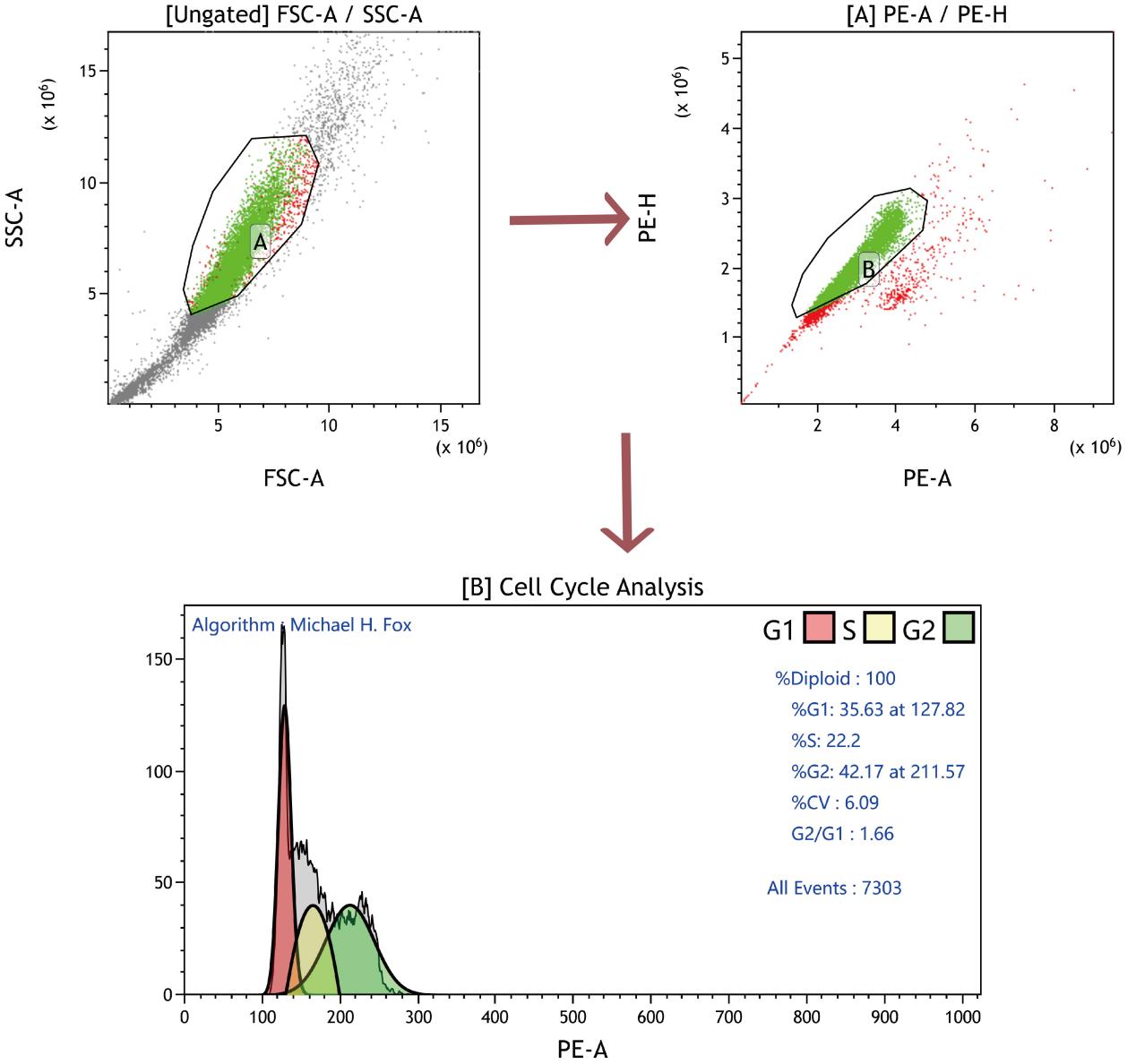


Supplementary Figure 1 The analysis strategy of flow cytometry. First, the single-cell population was identified and gated based on the FSC-A and SSC-A scatter plot (P1, denoted as A in the figure). Subsequently, debris and cell aggregates were excluded by gating on PE-A versus PE-H (P1, denoted as B in the figure). Finally, fluorescence intensity analysis was performed by plotting a histogram of PE-A expression within the P2-gated population.
